# Genetic Parameters, Prediction of Genotypic Values, and Forage Stability in *Paspalum nicorae* Parodi Ecotypes via REML/BLUP

**DOI:** 10.3390/genes16101164

**Published:** 2025-10-01

**Authors:** Diógenes Cecchin Silveira, Annamaria Mills, Júlio Antoniolli, Victor Schneider de Ávila, Maria Eduarda Pagani Sangineto, Juliana Medianeira Machado, Roberto Luis Weiler, André Pich Brunes, Carine Simioni, Miguel Dall’Agnol

**Affiliations:** 1 Faculdade de Agronomia, Universidade Federal do Rio Grande do Sul, Av. Bento Gonçalves 7712, Porto Alegre 90001-970, RS, Brazil; jl.antoniolli@hotmail.com (J.A.); victorneideravila@gmail.com (V.S.d.Á.); dudasangineto@gmail.com (M.E.P.S.); robertoluisw@yahoo.com.br (R.L.W.); andre.brunes@gmail.com (A.P.B.); carine.simioni@ufrgs.br (C.S.); mdallagnol@gmail.com (M.D.); 2 Field Research Centre, Lincoln University, P.O. Box 85084, Lincoln 7647, New Zealand; anna.mills@lincoln.ac.nz; 3 Departamento de Zootecnia, Centro de Ciências Rurais, Universidade Federal de Santa Maria, Av. Roraima nº 1000, Santa Maria 97105-900, RS, Brazil; juliana.machado@ufsm.br

**Keywords:** Brunswick grass, genetic biometrics, ideotype, MGIDI, plant breeding

## Abstract

Background/Objectives: *Paspalum nicorae* Parodi is a native subtropical grass species with promising agronomic attributes, such as persistence, drought and cold tolerance, and rapid establishment. However, the species remains underutilized in breeding programs due to the absence of well-characterized germplasm and limited studies on its genetic variability and agronomic potential. This study aimed to estimate genetic parameters, predict genotypic values, and identify superior ecotypes with desirable forage traits, integrating stability and adaptability analyses. Methods: A total of 84 ecotypes were evaluated over three consecutive years for twelve morphological and forage-related traits. Genetic parameters, genotypic values, and selection gains were estimated using mixed models (REML/BLUP). Stability was assessed through harmonic means of genotypic performance, and the multi-trait genotype–ideotype distance index (MGIDI) was applied to identify ecotypes with balanced performance across traits. Results: Substantial genetic variability was detected for most traits, particularly those related to biomass accumulation, such as total dry matter, the number of tillers, fresh matter, and leaf dry matter. These traits exhibited medium to high heritability and strong potential for selection. Ecotype N3.10 consistently showed superior performance across productivity traits while other ecotypes, such as N4.14 and N1.09, stood out for quality-related attributes and cold tolerance, respectively. The application of the MGIDI index enabled the identification of 17 ecotypes with balanced multi-trait performance, supporting the simultaneous selection for productivity, quality, and adaptability. Comparisons with *P. notatum* suggest that *P. nicorae* harbors competitive genetic potential, despite its lower level of domestication. Conclusions: The integration of REML/BLUP analyses, stability parameters, and ideotype-based multi-trait selection provided a robust framework for identifying elite *P. nicorae* ecotypes. These findings reinforce the strategic importance of this species as a valuable genetic resource for the development of adapted and productive forage cultivars in subtropical environments.

## 1. Introduction

The grassland and savanna ecosystems of South America harbor substantial biodiversity and play a fundamental role in sustaining ecosystem services. Functions include carbon sequestration, soil conservation, and hydrological cycle regulation [1]. These environments support animal production systems based on native pastures, which are essential for extensive livestock farming, particularly in tropical and subtropical regions.

The C_4_ genus *Paspalum* represents a crucial component of the grasslands and includes tropical and subtropical grasses with an essentially Pan-American distribution [2]. Approximately 350 species have been identified [3], of which 217 (62%) are found in Brazil [4]. This makes it the largest genus within the subfamily Panicoideae [5]. Its center of diversity is located in the Brazilian Cerrado, and its presence extends to the grasslands of southern Brazil, Argentina, and Uruguay [6,7,8]. The genus includes a high number of species endemic to Brazil. Many of these are restricted to specific biomes such as the Andes, Cerrado, the eastern coast of the United States, and the Caribbean [8]. Only a few species have been identified outside the Americas, with three or four considered cosmopolitans [6,9,10].

Among the species of agronomic interest, *P. nicorae* Parodi (Brunswickgrass) has previously been recognized for its forage potential [11]. In Brazil, its geographical distribution includes the Central-West (Mato Grosso do Sul) and Southern regions (Paraná, Santa Catarina, and Rio Grande do Sul) and occurs throughout phytogeographic domains of the Cerrado, Atlantic Forest, Pampa, and Pantanal [12]. Its frequent presence in sandy soils suggests tolerance to water deficit, which points to its potential suitability for use in drier environments. This set of attributes positions *P. nicorae* as a promising candidate species for breeding programs aimed at the restoration of degraded areas. Regardless, to date, there has been little work to breed improved forage cultivars from existing ecotypes.

The development of forage cultivars is a complex and long-term process that requires repeated evaluations over multiple seasons and years. Such programs demand continuous investment in research to identify and select superior genotypes that are more productive, resilient, and adapted to local environmental conditions [13]. Although precise estimates may vary, the cost of developing and releasing a new forage cultivar is substantial, underscoring the need for more efficient breeding strategies. Consequently, multi-trait selection techniques that reduce the time and number of cycles required for cultivar release offer not only genetic gains but also significant cost savings. These efforts are crucial to meet the growing demand for high-quality forage while ensuring the conservation of genetic resources.

Recent studies have indicated *P. nicorae* as a promising alternative to overcome the limitations of native and introduced pastures [14]. However, selection within the genus *Paspalum* has largely relied on field-based phenotypic evaluations combined with conventional statistical approaches [15,16,17,18,19], which limits accuracy in identifying superior genotypes. With advances in biometrics, linear mixed-effects models (LMMs) have become widely adopted to analyze data from genetic experiments. The benefit of this approach includes their ability to model random effects and more realistically represent genotype-by-environment (G × E) variation [20]. Within this framework, methodologies based on restricted maximum likelihood (REML) and best linear unbiased prediction (BLUP) have previously been successfully applied to bahiagrass (*P. notatum*), which has enabled the precise estimation of genetic parameters and accurate prediction of genotypic values [21,22,23]. Methodological comparisons have shown that BLUP outperforms classical models, such as additive main effect and multiplicative interaction (AMMI), because it has greater predictive accuracy and allows the inclusion of random environmental effects, which optimizes the analysis of G × E interactions [24]. The widespread adoption of this approach has been facilitated by the implementation of dedicated software routines for plant breeding applications [25,26].

Previous studies on *P. notatum* have demonstrated broad genetic variability and high genetic control over forage traits, which has led to significant advances in the selection of superior genotypes [23,24,25]. However, *P. nicorae* remains considerably less studied. This is partly due to the predominance of environments already well served by *P. notatum*, which has established itself as a resilient and broadly adapted species in subtropical regions of South America. In contrast, *P. nicorae* is naturally adapted to drier and sandier soils, making it a promising alternative or complementary option for marginal environments where *P. notatum* may not perform as well. Despite these valuable agronomic attributes, in some regions such as the United States, *P. nicorae* has been reported as having invasive potential. Furthermore, the lack of consolidated information about its genetic diversity and the scarcity of robust estimates of genetic parameters hinder its inclusion in breeding programs. This constitutes a substantial knowledge gap and limits progress in identifying and recommending superior genotypes for multiple agronomic traits under less favorable conditions.

This knowledge gap highlights the need for studies that quantify the genetic parameters of *P. nicorae*. The information gathered will allow the selection of superior genotypes capable of improved forage yield and stability, as well as enhanced seed production and quality. We hypothesize that significant genetic variability exists among ecotypes of *P. nicorae* and that the use of mixed models combined with ideotype-based multi-trait indices will enable the identification of elite genotypes with superior forage traits, stability, and adaptability for breeding purposes. Therefore, the objective of this study was to estimate genetic parameters and predict genetic gains in 84 *P. nicorae* ecotypes using the REML/BLUP approach. Additionally, the study evaluated stability and adaptability indices over three consecutive years of forage production. Multi-trait indices, such as MGIDI, were employed to identify material with superior genetic values, aiming to support the development of productive, stable, and well-adapted forage cultivars.

## 2. Materials and Methods

### 2.1. Location and Research Period

A field experiment (30°05′ S, 51°40′ W; 46 m a.s.l.) was conducted from November 2020 to February 2023 at the Agronomic Experimental Station of the Federal University of Rio Grande do Sul (UFRGS), in southern Brazil, within an area managed by the Department of Forage Plants and Agrometeorology (DPFA). The soil in the experimental area is classified as “Argissolo Vermelho Distrófico” (Ultisols; USDA Soil Taxonomy) [27], and the regional climate is classified as Cfa according to Köppen’s classification [28]. The mean annual precipitation is approximately 1450 mm [29]. Over a 50-year period (1969–2019), the average monthly maximum air temperature was 30.2 °C (January), and the minimum was 8.5 °C in July (Table 1).

### 2.2. Plant Material

We evaluated 84 ecotypes of *P. nicorae* Parodi collected from four physiographic regions in Rio Grande do Sul, Brazil in 2018. These ecotypes are part of the Germplasm Bank of the UFRGS Forage Plant Breeding Program [30]. The ecotypes were sampled in representative areas of the Pampa Biome, with a focus on areas with sandy soils predominantly found in native grassland regions where the target species is most abundant. Collection points were spaced ~10 km apart based on the natural distribution of the species across different physiographic regions. At each site, three to eight individuals per ecotype were collected to ensure coverage of various phenological stages. The collection process included recording geographic coordinates (GPS) and standardized morphological descriptors.

The collected plants were cultivated in 1.0 L pots to allow further development and observation. Environmental data, including soil type, associated vegetation, and climatic characteristics, were documented for subsequent analysis. When available, seeds were stored in hermetically sealed containers for preservation.

### 2.3. Site Preparation and Soil Fertility

In October 2020, the experimental area was sprayed with herbicide. Soil samples (0–0.2 m) were collected and analyzed prior to sowing, and fertilizer amendments were made based on guidelines of the Soil Chemistry and Fertility Commission of RS/SC [31]. At the end of each growth season, soil analysis was repeated (Table 2).

The experimental area was initially amended with 3.4 t/ha of dolomitic limestone (PRNT 80%) and partially fertilized with an 8-20-20 (N-P_2_O_5_-K_2_O) formula. In the first year, 950 kg ha^−1^ of fertilizer was applied, followed by 650 kg ha^−1^ in Years 2 and 3. This applied the equivalent of 76 kg N ha^−1^, 190 kg P_2_O_5_ ha^−1^, and 190 kg K_2_O ha^−1^ in Year 1 and 52 kg N ha^−1^, 130 kg P_2_O_5_ ha^−1^, and 130 kg K_2_O ha^−1^ in Years 2 and 3. Maintenance fertilizers were applied in split applications after each harvest, following technical recommendations for warm-season perennial grasses [31].

### 2.4. Seed Germination, Plant Establishment, and Experimental Design

*P. nicorae* ecotype seeds were obtained in 2019 from the Germplasm Bank of the UFRGS Forage Plant Breeding Program. In early August 2020, seeds were germinated by placing them on filter paper moistened with a 0.2% KNO_3_ solution inside plastic germination boxes (gerboxes) [32]. The boxes were maintained in a growth chamber under controlled conditions of 16 h of light and 8 h of darkness at a constant temperature of 30 °C for 28 days [32].

After the first leaf emerged, seedlings were transferred to plant tubes filled with Carolina Soil™ commercial substrate, which consists of peat, vermiculite, organic residue, and limestone. The seedlings were cultivated in the tubes until they reached the appropriate stage for transplantation. On 25 November 2020, seedlings were field sown using a manual planter. The experiment was arranged in a randomized complete block design (RCBD) with four replicates. The experimental units consisted of individual plants spaced 1 m apart within and between rows. Throughout the experimental period, the area was kept weed-free to ensure optimal plant development.

### 2.5. Evaluated Traits

Measurements included the following: number of tillers (NT, tillers plant^−1^), fresh mass (FM, g plant^−1^), leaf dry mass (LDM, g plant^−1^), stem dry mass (SDM, g plant^−1^), inflorescence dry mass (IDM, g plant^−1^), total dry mass (TDM, g plant^−1^), leaf-to-stem ratio (LSR), harvest index (HI), cold tolerance (CT), forage persistence (FP), growth habit (GH), and plant height (PH, cm).

Harvests were triggered whenever plant height reached 30 ± 2.5 cm. Samples were cut to a 10 cm residual height. In the first year, two cuts were conducted (15 January 2021 and 19 February 2021). In Year 2, six cuts were performed (28 October 2021, 20 November 2021, 22 December 2021, 18 January 2022, 5 February 2022, and 7 March 2022). Finally, in Year 3, five cuts were carried out (1 November 2022, 30 November 2022, 23 December 2022, 16 January 2023, and 19 January 2023).

At each harvest date, NT per plant was counted manually. After cutting, samples were separated morphologically into leaves (leaf blades), stems (stems plus sheaths), and inflorescences, and dried in a forced-air oven at 65 °C until constant weight. LDM, SDM, and IDM were measured individually, and their sum was used to compute TDM. The LSR was calculated as LDM/SDM, and HI as LDM/TDM. GH was visually assessed at each cut and classified into four categories: 1 = erect, 2 = semi-erect, 3 = semi-prostrate, and 4 = prostrate. PH was measured using a graduated ruler from ground level to the tip of the tallest leaf. During winter, CT was evaluated on a 1–5 scale based on the degree of cold damage observed: 1 = fully senesced (highly susceptible) and 5 = fully green (highly tolerant). FP was assessed at the end of the final production cycle based on plant survival, where each plant was scored as 1 if alive or 0 if dead, and the mean score per genotype was used to estimate persistence across years.

### 2.6. Statistical Analysis

#### 2.6.1. REML/BLUP

Estimates of variance components and genetic parameters, as well as the prediction of genotypic values, were obtained using the REML/BLUP method, along with the analysis of genotypic stability based on the harmonic mean of genotypic values (MHVG). The significance of random effects was assessed through deviance analysis (ANADEV) based on the likelihood ratio test, using a chi-square distribution with one degree of freedom and a 5% significance level [33].

The statistical model employed was appropriate for randomized complete block designs conducted at a single location, across multiple harvests, with one observation per plot. Thus, the model used was as follows:

$$y=X_{m}+Z_{g}+W_{p}+T_{i}+e$$

in which *y* is the data vector; *m* is the vector of measurement–replication combination effects (assumed as fixed), added to the grand mean; *g* is the vector of genotypic effects (assumed as random); *p* is the vector of permanent environmental effects (i.e., plots, assumed as random); *i* is the vector of genotype × measurement interaction effects (random); and *e* is the residual vector (random). The uppercase letters X, Z, W, and T represent the incidence matrices for the corresponding effects.

The mixed-model equations can be represented as follows:

$$\left[\begin{array}{cccc} X^{\prime }X & X^{\prime }Z & X^{\prime }W & X^{\prime }T\\ Z^{\prime }X & Z^{\prime }Z+I^{-1\lambda }1 & Z^{\prime }W & Z^{\prime }T\\ W^{\prime }X & W^{\prime }Z & W^{\prime }W+\;I^{-1\lambda }2 & W^{\prime }T\\ T^{\prime }X & T^{\prime }Z & T^{\prime }W & T^{\prime }T+\;I^{-1\lambda }3 \end{array}\right]\left[\begin{array}{c} \tilde{m}\\ \tilde{g}\\ \tilde{p}\\ \tilde{i} \end{array}\right]=\left[\begin{array}{c} X^{\prime }y\\ Z^{\prime }y\\ W^{\prime }y\\ T^{\prime }y \end{array}\right]$$

where

$$\lambda_{1}=\frac{1-\rho }{h^{2}}=\frac{{\hat{\sigma }}_{e}^{2}}{{\hat{\sigma }}_{g}^{2}};\;\lambda_{2}=\frac{1-\rho }{c^{2}};\;\lambda_{3}=\frac{1-\rho }{p^{2}}=\frac{{\hat{\sigma }}_{e}^{2}}{{\hat{\sigma }}_{p}^{2}}$$

The individual broad-sense heritability within blocks is given thus:

$$h^{2}=\frac{{\hat{\sigma }}_{g}^{2}}{{\hat{\sigma }}_{g}^{2}+{\hat{\sigma }}_{c}^{2}+{\hat{\sigma }}_{p}^{2}+{\hat{\sigma }}_{e}^{2}}$$

The individual repeatability within blocks is given thus:

$$\rho =\frac{{\hat{\sigma }}_{g}^{2}+{\hat{\sigma }}_{c}^{2}+{\hat{\sigma }}_{p}^{2}}{{\hat{\sigma }}_{g}^{2}+{\hat{\sigma }}_{c}^{2}+{\hat{\sigma }}_{p}^{2}+{\hat{\sigma }}_{e}^{2}}$$

The coefficient of determination of the permanent plot effects is given thus:

$$P^{2}=\frac{{\hat{\sigma }}_{p}^{2}}{{\hat{\sigma }}_{g}^{2}+{\hat{\sigma }}_{c}^{2}+{\hat{\sigma }}_{p}^{2}+{\hat{\sigma }}_{e}^{2}}$$

The common environmental correlation between plots is given thus:

$$c^{2}=\frac{{\hat{\sigma }}_{c}^{2}}{{\hat{\sigma }}_{g}^{2}+{\hat{\sigma }}_{c}^{2}+{\hat{\sigma }}_{p}^{2}+{\hat{\sigma }}_{e}^{2}}$$

The iterative estimators of the variance components in REML were obtained using the Expectation-Maximization (EM) algorithm [34].

$${\hat{\sigma }}_{e}^{2}=\left[y^{\prime }y-\hat{m^{\prime }}X^{\prime }y-{\hat{g}}^{\prime }Z^{\prime }y-{\hat{p}}^{\prime }W^{\prime }y-{\hat{i}}^{\prime }T^{\prime }y\right]/\left[N-r(X)\right]$$

$${\hat{\sigma }}_{g}^{2}=\left[{\hat{g}}^{\prime I^{-1}}\hat{g\;}+{\hat{\sigma }}_{e}^{2}\;\mathrm{tr}(I^{-1}C^{22}\right]/q$$

$${\hat{\sigma }}_{p}^{2}=\left[{\hat{p}}^{\prime }p+{\hat{\sigma }}_{e}^{2}\;\mathrm{tr}{\;C}^{33}\right]/s$$

$${\hat{\sigma }}_{i}^{2}=\left[{\hat{i}}^{\prime }i+{\hat{\sigma }}_{e}^{2}\;\mathrm{tr}{\;C}^{44}\right]/q$$

where $C^{22},\;{\;C}^{33},$ and ${\;C}^{44}$ come from the following:

$$C^{-1}={\left[\begin{array}{cccc} C_{11} & C_{12} & C_{13} & C_{14}\\ C_{21} & C_{22} & C_{23} & C_{24}\\ C_{31} & C_{32} & C_{33} & C_{34}\\ C_{41} & C_{42} & C_{43} & C_{44} \end{array}\right]}^{-1}=\left[\begin{array}{cccc} C^{11} & C^{12} & C^{13} & C^{14}\\ C^{21} & C^{22} & C^{23} & C^{24}\\ C^{31} & C^{32} & C^{33} & C^{34}\\ C^{41} & C^{42} & C^{43} & C^{44} \end{array}\right]$$

where C = coefficient matrix of the mixed-model equations; tr = trace operator; r(X) = rank of matrix X; N = total number of observations; q = number of individuals; s = number of ecotype × harvest combinations.

The variance components associated with the model effects are as follows:

${\hat{h}}_{g}^{2}=\frac{{\hat{\sigma }}_{g}^{2}}{{\hat{\sigma }}_{f}^{2}}$ = Broad-sense heritability of individual plots, that is, of the total genotypic effects;

$C_{p}^{2}$ = Coefficient of determination of the plot effects;

$C_{gm}^{2}=\frac{{\hat{\sigma }}_{\mathrm{int}}^{2}}{{\hat{\sigma }}_{f}^{2}}$ = Coefficient for determining the effects of the genotypes x measurements interaction;

$r_{gmed}=\frac{{\hat{\sigma }}_{g}^{2}}{{(\hat{\sigma }}_{g}^{2}+{\hat{\sigma }}_{\mathrm{int}}^{2})\;}$ = Genotypic correlation through measurements;

$h_{\mathrm{mg}}^{2}=\;\frac{{\hat{\sigma }}_{g}^{2}}{{\hat{\sigma }}_{g\;}^{2}+\left(\frac{{\hat{\sigma }}_{e}^{2}}{b}\right)+\left(\frac{{\hat{\sigma }}_{c}^{2}}{\mathrm{nb}}\right)\;}$ = Heritability of the genotype mean; n: number of plots; b: number of blocks;

${\hat{r}}_{\mathrm{gg}}=\sqrt{h_{mg}^{2}}$ = Accuracy of genotype selection;

${\mathrm{CV}}_{g}(\%)=\frac{\sqrt{{\hat{\sigma }}_{g}^{2}}}{\mu }*100$ = Genotypic coefficient of variation;

${\mathrm{CV}}_{e}(\%)=\frac{\sqrt{{\hat{\sigma }}_{c\;}^{2}}}{\mu }*100$ = Environmental coefficient of variation;

${\mathrm{CV}}_{r}(\%)=\frac{{\mathrm{CV}}_{g}}{{\mathrm{CV}}_{e}}$ = Relative coefficient of variation.

The variation index (VI), as described by Pimentel-Gomes [35], can be calculated for both environmental and genotypic components by dividing the corresponding coefficient of variation (CV_e_ or CV_g_) by the square root of the number of replications. This index serves as a measure of precision and relative variability, where lower VI_e_ values indicate greater experimental reliability while higher VI_g_ values suggest a favorable scenario for genotype selection due to higher genetic variability in relation to sampling error.

$${VI}_{e}=VIe\frac{{CV}_{e}}{\sqrt{r}}$$

$${VI}_{g}=\frac{{CV}_{g}}{\sqrt{r}}$$

For each trait, the response variable used in the analysis corresponded to the mean value across all harvests conducted during the evaluation period. Thus, the reported grand mean (µ) represents the average performance of each genotype over all measurement events.

#### 2.6.2. Selection Index

The selection gain (SG, %) obtained through selection among ecotypes was calculated as follows:

$$\mathrm{SG}\;(\%)\;=\;100\;\times \;\frac{{\mathrm{GS}}_{s}-{\mathrm{OG}}_{m}}{{\mathrm{OG}}_{m}}$$

where SG is the genotypic mean of the selected ecotypes and OG_m_ is the grand genotypic mean.

Productive stability was assessed using the harmonic mean of genotypic values (MHVG):

$$\mathrm{MHV}G_{i}\;=\;n/\sum_{j=1}^{N}\left(1/{Vg}_{ij}\right)$$

where n is the number of environments (n = 13) in which ecotype is i and ${Vg}_{ij}$ is the genotypic value of ecotype i in environment j, expressed as a proportion of the environmental mean [36].

Productive adaptability was assessed through the relative performance of genotypic values (PRVG), calculated as follows:

$$\mathrm{PRV}G_{i}=n/\sum_{j=1}^{N}\left(1/{Vg}_{ij}\right),$$

Finally, the joint selection index, simultaneously considering forage productivity, stability, and adaptability (MHPRVG), was calculated as follows:

$$\mathrm{MHPRV}G_{i}=n/(\sum_{j=1}^{N}\times \;1)/{Vg}_{ij}$$

The mean genotypic value capitalizing adaptability is obtained by multiplying RPGV by the grand mean across all environments (RPGV × µ) while the mean genotypic value penalized by instability and capitalized by stability is calculated by multiplying HMRPGV by the grand mean across all environments (HMRPGV × µ).

#### 2.6.3. Multi-Trait Genotype–Ideotype Distance Index

To calculate the multi-trait genotype–ideotype distance index (MGIDI) [37], the ideotype was defined based on the primary goals of most forage grass breeding programs, which typically aim to improve traits associated with biomass production and plant vigor. Accordingly, the ideotype was constructed to favor higher values for NT, FM, LDM, TDM, LSR, CT, GH, and PH and lower values for SDM and IDM. The traits HI and FP were not included in the ideotype definition due to the absence of significant genetic variation. A selection intensity of 15% was applied and resulted in the selection of 18 ecotypes out of a total of 84.

The MGIDI was calculated following the four-step procedure proposed by Olivoto and Nardino [37]: (1) trait rescaling to a 0–100 range, where the maximum value is set to 100 and the minimum to 0 when higher trait values are desired, and vice versa when lower values are preferred; (2) exploratory factor analysis of the rescaled data to group correlated traits into latent factors and to compute factor scores for each genotype; (3) construction of the ideotype based on the optimal values (i.e., 100) for all traits under selection; and (4) calculation of distance between each genotype and the ideotype in the factor space. The distance between genotype *i* and the ideotype was computed using the following equation:

$${MGIDI}_{i}=\sqrt{\sum_{j=1}^{f}{\left(F_{ij}-F_{j}\right)}^{2}}$$

where MGIDI_i_ is the multi-trait genotype–ideotype distance index for the *i*th genotype; *F_ij_* is the score of the *i*th genotype in the *j*th factor (*i* = 1, 2,… *g*; *j* = 1, 2,…, *f*), where *g* and *f* represent the numbers of genotypes and factors, respectively. *F_j_* is the *j*th score of the ideotype. The genotype with the lowest MGIDI is then closer to the ideotype and, therefore, should present desired values for all 10 traits analyzed [37].

All data were analyzed using R software (version 4.3.2) [38], along with the metan package (version 1.20.0) [26], which has been specifically developed for multivariate and mixed-model analyses in plant breeding. Additional analyses were conducted using SELEGEN-REML/BLUP [39] and GENES software (version 2023.1) [40], both widely used for genetic parameter estimation and selection in perennial species.

## 3. Results

### 3.1. Estimates of Variance Components via REML

Most of the evaluated traits were affected (*p* < 0.01) by genotypic, environmental, and genotype × environment interaction effects (Table 3). However, the traits HI and FP did not exhibit genotypic effects (*p* > 0.01), indicating limited genetic variability in the evaluated population. The results indicated the presence of genetic variability for the majority of the traits (NT, FM, LDM, SDM, IDM, TDM, LSR, CT, GH, and PH), allowing for more efficient selection. The G×E interaction confirmed that genotypes performed differently depending on the evaluation method, highlighting the importance of multi-environment trials for identifying stable and adapted genotypes.

The analysis of the CV_e_ and CV_g_ values revealed marked differences among the traits. For example, CV_e_ ranged from 11.73% (NT) to 51.45% (IDM). The values indicated greater experimental precision for NT and higher environmental variability for IDM. The CV_e_ values for GH (15.76%) and PH (13.32%), indicated measurement precision over time (Table 3). Genotypic coefficients of variation (CV_g_) ranged from 0.99% (LSR) to 19.37% (GH). The highest CV_g_ values were observed for NT (17.51%), FM (14.39%), and GH (19.37%).

The VI_e_ and VI_g_ variation indices followed a similar pattern. The environmental variation index (VI_e_) ranged from 5.87% (NT) to 25.73% (IDM) while VI_g_ ranged from 0.50% (LSR) to 9.69% (GH). The GH (9.69%) and NT (8.76%) traits exhibited the highest VI_g_ values, which reinforces their relevance for breeding programs. The relative variation coefficient (CV_r_) was 1.49 for NT and 1.23 for GH. Values >1 indicate high selection efficiency for these traits.

### 3.2. Genetic Parameters

**Figure 1:**
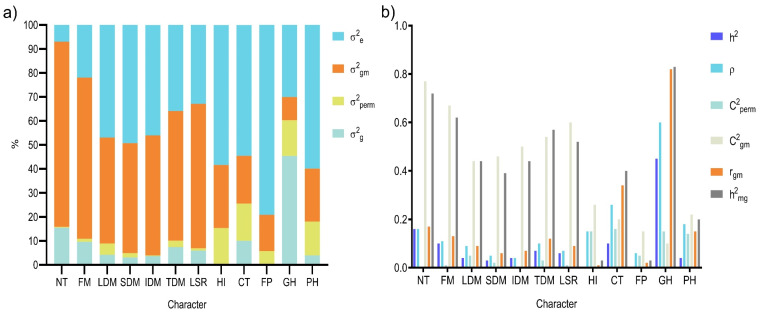
Decomposition of variance components (**a**) and estimation of genetic parameters (**b**) for 12 forage traits in 84 *P. nicorae* Parodi ecotypes using a mixed model approach. $\sigma_{e}^{2}$: environmental variance; $\sigma_{gm}^{2}$: genotype-by-measurement interaction variance; $\sigma_{perm}^{2}$: permanent environment variance; $\sigma_{g}^{2}$: genotypic variance. h^2^: broad-sense heritability of individual plots, considering total genotypic effects; ρ: repeatability at the plot level; $C_{perm}^{2}$ = coefficient of determination of plot effects; $C_{gm}^{2}$= coefficient for determining the effects of the genotypes x measurements interaction; $r_{gmed}$ = genotypic correlation through measurements; $h_{\mathrm{mg}}^{2}=$ heritability of the genotype mean. NT: number of tillers; FM: fresh matter; LDM: leaf dry matter; SDM: stem dry matter; IDM: inflorescence dry matter; TDM: total dry matter; LSR: leaf/stem ratio; HI: harvest index; CT: cold tolerance; FP: forage persistence; GH: growth habit and PH: plant height.

The decomposition of variances (Figure 1a) revealed that genotypic variance (${\hat{\sigma }}_{g}^{2}$) was highest for GH (45.38%), NT (15.53%), CT (10.04%), FM (9.65%), and TDM (7.42%), which indicated that there was a strong genetic component to the expression. In contrast, HI (0.24%) and FP (0.37) exhibited low ${\hat{\sigma }}_{g}^{2}$, values, suggesting limited genetic variability and weak genetic control over these traits in the studied population. Environmental variance (${\hat{\sigma }}_{e}^{2}$) was most pronounced for FP (79.08%), PH (59.91%), HI (58.38%), SDM (49.27%), LDM (46.97%), and IDM (46.05%), which appeared to indicate a greater influence of environmental factors on these traits. Traits such as NT (6.97%), FM (21.96%), and GH (30.01%) had lower ${\hat{\sigma }}_{e}^{2}$ values, which enhanced the efficiency of genetic selection.

The variance associated with the genotype × measurement interaction (${\hat{\sigma }}_{gm}^{2}$) was highest for NT (77.18%), FM (67.11%), and LSR (60.19%), which suggested that the genotypic stability of these traits is strongly affected by repeated measurements. The permanent environmental variance (${\hat{\sigma }}_{perm}^{2}$) was 15.52% for CT, 15.17% for HI, 14.92% for GH, and 14.08% for PH, indicating that persistent environmental effects may impact the phenotypic stability of these traits over time.

The estimates of genetic parameters revealed varying levels of genetic control across traits evaluated in the germplasm studied (Figure 1b). The broad-sense heritability of individual plots (h^2^) ranged from 0.002 for HI to 0.454 for GH. This result showed that there was greater genetic control over GH in contrast to a strong environmental influence on HI. The CT and LSR traits presented intermediate heritability values of 0.100 and 0.060, respectively (Figure 1b).

Repeatability at the plot level (ρ) varied considerably. Values ranged from a minimum of 0.058 for FP to a maximum of 0.603 for GH, which showed that there was high genetic consistency for GH and the extent of environmental susceptibility for FP. The coefficient of determination of the permanent plot effects ($C_{perm}^{2}$) was low for most traits and ranged from 0.003 (NT and IDM) to 0.047 (LDM). This showed that these effects contributed minimally to phenotypic variation.

The genotype × measurement interaction, quantified by the coefficient of determination for this interaction ($C_{gm}^{2}$), ranged from 0.097 for GH to 0.602 for LSR. This result suggested a greater interaction effect for LSR compared with GH. The genotypic correlation across measurements ($r_{gmed}$) was highest for GH (0.603) and lowest for FP (0.058). A high $r_{gmed}$ reflects genetic stability over time and a low value indicates that the result was more affected by environmental and temporal factors.

Finally, the heritability of genotypic means ($h_{\mathrm{mg}}^{2}$) ranged from 0.151 for FP to 0.541 for TDM. The higher value for TDM indicated that there was greater potential for selective gains while traits with low values, such as FP, would require greater attention to ensure genetic stability in future selection cycles.

### 3.3. Genotypic Values and Genetic Gains

The evaluation of average genotypic values (u + g + gem) and selection gains (SG%) highlighted the superiority of specific ecotypes for the 12 assessed traits. Full details of the ranking of the 84 ecotypes for the 10 evaluated traits are shown in Appendix A. At the extremes were Ecotypes N3.10 and N4.17. Ecotype N3.10 ranked first of 84 ecotypes in six traits (NT, FM, LDM, SDM, IDM, and TDM) and was within the top 10 for PH. It ranked 59th for LSR, 81st for GH, and 84th for GH. At the other end of the rankings, Ecotype N4.17 ranked last in FM, LDM, SDM, and TDM. It also ranked 83rd for NT and 82nd for IDM, which was likely a function of the low ranking for NT. For LSR and CT, the ranks were 72nd and 69th, respectively. The highest rank achieved by Ecotype N4.17 was 12th for GH. Ecotypes which fell within the top 10 for each of the 10 traits analyzed by BLUP follow:

For NT, Ecotype N3.10 had the highest mean (375.99 g plant^−1^) and the SG% (58.45%). Other ecotypes, within the top 10, for NT included N3.17 (331.38; SG: 42.93%), N3.12 (281.20; SG: 25.48%), N5.09 (275.51; SG: 23.50%), N2.11 (266.13; SG: 20.24%), N2.07 (258.97; SG: 17.74%), N8.04 (258.55; SG: 17.60%), N4.09 (258.44; SG: 17.56%), N5.01 (256.38; SG: 16.84%), and N3.18 (253.95; SG: 16.00%).

For FM, Ecotype N3.10 again ranked first (244.15 g plant^−1^; SG: 37.36%), which demonstrated its high productive potential. The top 10 ecotypes were rounded out by N2.06 (210.20, g plant^−1^; SG: 23.10%), N3.17 (204.78, g plant^−1^; SG: 20.83%), N4.09 (202.43, g plant^−1^; SG: 19.84%), N5.01 (200.23, g plant^−1^; SG: 18.92%), N1.21 (198.89, g plant^−1^; SG: 18.35%), N2.07 (190.27, g plant^−1^; SG: 14.74%), N2.11 (188.74, g plant^−1^; SG: 14.09%), N1.05 (187.98, g plant^−1^; SG: 13.77%), and N3.09 (187.98, g plant^−1^; SG: 13.77%).

For LDM, L3.10 once again exhibited the best performance of the 84 ecotypes evaluated (96.00, g plant^−1^; SG: 29.96%). The remaining top 10 productive ecotypes for this LDM included N2.06 (81.19, g plant^−1^; SG: 16.78%), N1.05 (79.77, g plant^−1^; SG: 15.52%), N3.17 (77.77, g plant^−1^; SG: 13.75%), N2.11 (75.30, g plant^−1^; SG: 11.55%), N5.01 (73.97, g plant^−1^; SG: 10.37%), N3.12 (73.47, g plant^−1^; SG: 9.92%), N3.18 (73.24, g plant^−1^; SG: 9.71%), N8.04 (72.47, g plant^−1^; SG: 9.03%), and N1.06 (72.34, g plant^−1^; SG: 8.92%).

For SDM, Ecotype L3.10 remained in prominence (10.69, g plant^−1^; SG: 29.65%), followed by L3.17 (9.53, g plant^−1^; SG: 21.31%), L2.06 (9.17, g plant^−1^; SG: 18.77%), L3.05 (7.88, g plant^−1^; SG: 9.52%), L6.22 (7.86, g plant^−1^; SG: 9.39%), L1.06 (7.84, g plant^−1^; SG: 9.26%), L1.21 (7.84, g plant^−1^; SG: 9.22%), L1.19 (7.70, g plant^−1^; SG: 8.29%), L3.04 (7.63, g plant^−1^; SG: 7.76%), and L3.09 (7.56, g plant^−1^; SG: 7.23%).

For IDM, L3.10 exhibited the highest mean (5.81, g plant^−1^; SG: 24.70%), followed by L3.18 (5.71, g plant^−1^; SG: 23.40%), L2.16 (5.30, g plant^−1^; SG: 18.21%), L2.07 (5.27, g plant^−1^; SG: 17.85%), L3.17 (5.22, g plant^−1^; SG: 17.19%), L3.04 (5.09, g plant^−1^; SG: 15.58%), L2.11 (5.07, g plant^−1^; SG: 15.29%), L4.07 (5.06, g plant^−1^; SG: 15.14%), L2.02 (5.01, g plant^−1^; SG: 14.58%), and L1.05 (4.86, g plant^−1^; SG: 12.66%).

Ecotype L3.10 also ranked first for TDM (125.44, g plant^−1^; SG: 41.84%) because it had the highest biomass accumulation across the three years of measurement. Nine other ecotypes also had notable production: L2.06 (99.69, g plant^−1^; SG: 20.09%), L3.17 (95.62, g plant^−1^; SG: 16.66%), L1.05 (92.70, g plant^−1^; SG: 14.19%), L1.21 (91.97, g plant^−1^; SG: 13.58%), L4.09 (91.37, g plant^−1^; SG: 13.07%), L3.18 (90.93, g plant^−1^; SG: 12.70%), L1.06 (89.43, g plant^−1^; SG: 11.43%), L5.01 (88.92, g plant^−1^; SG: 11.00%), and L6.22 (88.77, g plant^−1^; SG: 10.87%).

For LSR, Ecotype L4.14 ranked first (4.48, g plant^−1^; *SG*: 29.82%), followed by L4.10 (4.17, g plant^−1^; *SG*: 23.82%), L5.12 (4.03, g plant^−1^; *SG*: 21.15%), L1.13 (3.97, g plant^−1^; *SG*: 19.95%), L1.10 (3.87, g plant^−1^; *SG*: 18.00%), L7.20 (3.77, g plant^−1^; *SG*: 16.16%), L1.09 (3.76, g plant^−1^; *SG*: 15.81%), L2.01 (3.66, g plant^−1^; *SG*: 13.98%), L1.21 (3.63, g plant^−1^; *SG*: 13.41%), and L2.06 (3.61, g plant^−1^; *SG*: 12.96%).

For cold tolerance (CT), the highest mean value was obtained by L1.09 (4.39; *SG*: 27.14%), followed by these ecotypes: L1.13 (4.01; *SG*: 19.43%), L1.12 (3.73; *SG*: 13.93%), L1.07 (3.67; *SG*: 12.83%), L8.03 (3.51; *SG*: 9.53%), L1.14 (3.45; *SG*: 8.43%), L1.16 (3.45; *SG*: 8.43%), L7.15 (3.45; *SG*: 8.43%), L3.22 (3.40; *SG*: 7.32%), and L4.21 (3.40; *SG*: 7.32%).

Finally, for plant height (PH), Ecotype L2.02 presented the highest value (65.46, cm; *SG*: 5.21%), followed by L1.18 (62.96, cm; *SG*: 3.66%), L3.09 (61.89, cm; *SG*: 3.00%), L2.06 (61.88, cm; *SG*: 3.00%), L2.10 (60.77, cm; *SG*: 2.31%), L5.09 (60.38, cm; *SG*: 2.07%), L1.21 (60.32, cm; *SG*: 2.03%), L3.02 (60.20, cm; *SG*: 1.96%), L4.07 (59.94, cm; *SG*: 1.80%), and L3.10 (59.58, cm; *SG*: 1.58%).

The combination of medium to high heritability estimates and elevated genotypic variances for traits such as NT, GH, TDM, FM, and LDM underscores their strategic relevance for recurrent selection in *P. nicorae*. These traits are directly associated with agronomic attributes like canopy coverage, grazing tolerance, and biomass productivity, wich are key components of forage resilience in subtropical systems. Ecotype N3.10 stood out as the most promising for overall biomass accumulation, while N4.14 presented superior forage quality (high LSR), and N1.09 demonstrated high cold tolerance (high CT), making them priority candidates for targeted breeding. The variability among ecotypes offers opportunities to align selection with specific environmental conditions: for instance, N3.10 may be suitable for high-input systems whereas N1.09 has potential for cold-prone areas. These results reinforce the importance of tailoring selection strategies to local contexts, expanding the genetic base available for cultivar development, and increasing the adaptability and persistence of forage systems in marginal subtropical environments.

### 3.4. Adaptability, Stability, and Performance via BLUP-Based Indices

Estimates of genotypic stability, adaptability, and performance for *P. nicorae* ecotypes revealed considerable variability across the evaluated forage traits. Again, Ecotype N3.10 ranked highly (top three) in six of the ten traits. The results are reported in full in Appendix B. For the trait number of tillers (NT), the top five ecotypes identified were N3.10, N3.17, N1.21, N5.01, and N5.09. Like in the previous analysis, Ecotype N3.10 stood out with the highest values for MHVG (159.48), PRVG (2.40), PRVG*µ (499.68), MHPRVG (1.53), and MHPRVG*µ (319.02). The top five ecotypes for the FM trait were N3.10, N2.06, N4.09, N1.21, and N1.05, with N3.10 again ranking highest for MHVG (67.13), PRVG (1.79), PRVG*µ (278.14), MHPRVG (1.42), and MHPRVG*µ (220.60). These indices confirm the potential of these ecotypes in terms of genotypic stability and adaptability.

For LDM, Ecotypes N1.05, N2.06, N3.10, N5.01, and N4.09 were ranked top five. Ecotype N1.05 had the highest values for MHVG (34.42), PRVG (1.36), PRVG*µ (84.56), MHPRVG (1.28), and MHPRVG*µ (79.46) and established itself as the most promising breeding line for this trait.

For SDM, the top five ecotypes were N2.06, N3.10, N5.01, N3.17, and N3.05. Ecotype N2.06 produced the highest values of MHVG (4.86), PRVG (1.49), PRVG·µ (9.73), MHPRVG (1.40), and MHPRVG*µ (9.17), indicating strong stability and adaptability for this trait. For IDM, Ecotypes N3.10, N1.05, N1.21, N5.15, and N3.04 were the top performers. Ecotype N3.10 again achieved the highest MHVG (2.47), PRVG (1.84), PRVG*µ (7.12), MHPRVG (1.36), and MHPRVG*µ (5.24), which further reinforced its potential for this trait. Total dry matter (TDM) was superior from the N3.10, N2.06, N1.05, N1.21, and N4.09 ecotypes. Once again, N3.10 showed superior performance through its MHVG (41.40), PRVG (1.93), PRVG*µ (146.27), MHPRVG (1.43), and MHPRVG*µ (108.77) values.

The highest LSR values were observed in Ecotypes N1.13, N5.12, N1.21, N4.10, and N2.01. Among them, N1.13 exhibited the highest values for MHVG (3.13), PRVG (1.35), PRVG*µ (3.96), MHPRVG (1.28), and MHPRVG*µ (3.74), demonstrating greater efficiency in leaf blade accumulation relative to stems. For cold tolerance (CT), Ecotypes N1.09, N1.13, N1.12, N1.07, and N8.03 performed best, with N1.09 exhibiting the highest MHVG (4.35), PRVG (1.51), PRVG*µ (4.59), MHPRVG (1.45), and MHPRVG*µ (4.39).

Finally, for plant height (PH), Ecotypes N2.02, N1.18, N3.09, N2.06, and N2.10 presented the highest values. Ecotype N2.02 stood out with its MHVG (64.21), PRVG (1.15), PRVG*µ (64.46), MHPRVG (1.15), and MHPRVG*µ (65.37), reflecting its high vertical growth capacity. These results reinforce the superiority of Ecotypes N3.10 and N1.05, which repeatedly ranked in the top five for performance across multiple traits. These ecotypes demonstrate potential for use in breeding programs for increased yield, genotypic stability, and adaptation to diverse environmental conditions.

*P. nicorae* has been little studied and subjected to limited breeding efforts; however, the variability observed among ecotypes, particularly for traits related to biomass and persistence (e.g., TDM, NT, GH, and CT). Notably, Ecotype N1.09, which exhibited the highest cold tolerance score, exemplifies the presence of genotypes with specific adaptations that can expand the cultivation range of the species. This adaptive variability, coupled with the identification of ecotypes like N3.10 and N4.14 for productivity and quality, respectively, highlights the ecological relevance of *P. nicorae* as a complementary or alternative forage option in regions where traditional cultivars such as P. notatum may not perform optimally.

### 3.5. Multi-Trait Genotype–Ideotype Distance Index

**Figure 2:**
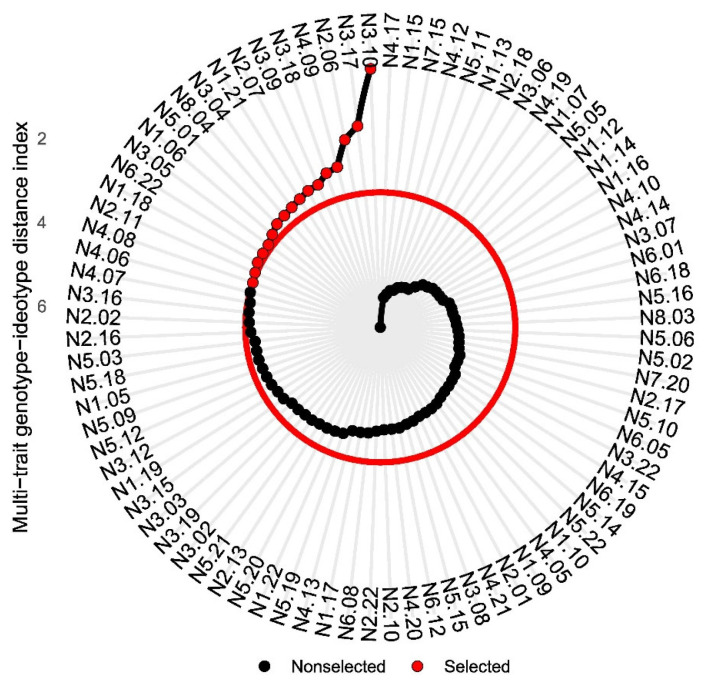
Classification and selection of 84 *P. nicorae* Parodi ecotypes using MGIDI. The spiral represents the ranking of genotypes based on the MGIDI values. Red points indicate the ecotypes selected under a 15% selection intensity (threshold represented by the red circle) while black points represent the non-selected ecotypes.

The application of the multi-trait genotype–ideotype distance index (MGIDI) allowed the classification of 84 *P. nicorae* ecotypes based on their proximity to the reference ideotype (Figure 2). A selection pressure of approximately 15% identified the 17 top-performing ecotypes: N3.10, N3.17, N2.06, N4.09, N3.18, N3.09, N2.07, N1.21, N3.04, N8.04, N5.01, N1.06, N3.05, N6.22, N1.18, N2.11, and N4.08.

## 4. Discussion

All traits, except for the harvest index (HI) and forage persistence (FP), showed significant genetic effects (Table 3). This provided evidence of the presence of genetic variability and, subsequently, the potential for selection gains in breeding programs. As expected, environmental effects were also significant for most traits. The exceptions were IDM and FP, which suggested lower environmental influence on these particular traits. The G × E interaction was significant for all 12 traits. This continues to reinforce the need for multi-environment trials, which has also been recommended for to enable effective selection.

Similar results were reported by Silveira et al. [23], who evaluated 195 *P. notatum* (bahiagrass) genotypes. They reported that six of the seven traits measured exhibited genetic variability and a significant G × E interaction. The exception was LSR. Likewise, Marcón et al. [21] identified significant genetic differences for most traits in *Paspalum* using the likelihood ratio test (LRT) at the 5% significance level, supporting the existence of genetic variability within the genus. These findings reinforce the importance of multi-environmental evaluation in selecting superior genotypes of *P. nicorae* (Brunswickgrass) because results must account for environmental effects and phenotypic stability to accurately identify the most promising ecotypes for future breeding programs.

The magnitude of the genotypic coefficient of variation (CV_g_) serves as an indicator of the genetic variability available for selection. Sebbenn et al. [41] previously suggested that CV_g_ values >7% are considered high. In the present study, all traits in *P. nicorae* exhibited CV_g_ values exceeding this threshold, except for HI (0.99%), FP (2.34%), and PH (3.44%), suggesting a high potential for genetic gain through selection. Previously, in *P. notatum*, CV_g_ values ranging from 27.86% (PH) to 78.70% (SDM) had been reported (Silveira et al., 2022) [22]. Similarly, values from 13.2% (GH) to 78.8% (LSR) had been observed in another population [23]. The values found in the present study for *P. nicorae* fell within the range reported for *Paspalum*, reinforcing the expressive genetic variability observed in *P. nicorae* ecotypes and their suitability for use in breeding programs.

The high genetic variability evidenced by CV_g_ suggests strong potential for selection response in most traits studied. However, to maximize selection efficiency, it is essential to evaluate the relationship between genetic and environmental variation. The relative coefficient of variation (CV_r_) supports this interpretation, where values >1.0 indicate that genetic variability exceeds environmental variability, thereby favoring the selection of superior genotypes [42,43]. The relative coefficient of variation (CV_r_), when analyzed alongside heritability estimates, helps determine the reliability of phenotypic values in representing genotypic performance [44]. In the present study, only NT (1.49) and GH (1.23) exhibited CV_r_ values above this threshold, which indicates stronger genetic control and greater potential for genetic gains in these traits (Table 3). In contrast, traits such as HI, FP, and PH, which also had a low CV_g_, had CV_r_ values below 1.0. This indicated a lower contribution of genetic variance and a greater influence of environmental factors on their expression for these three traits.

In the present study, several *P. nicorae* traits presented CV_r_ values below 1.0, suggesting a stronger environmental influence on phenotypic expression. In contrast, the evaluation of 74 *P. notatum* hybrids and three parent lines by Silveira et al. [22] reported CV_r_ values greater than 1.0 for all traits evaluated. This indicated that genetic variability had a greater impact on the observed traits than environmental variability. In another study, on a different population of *P. notatum* hybrids, only plant height exceeded a threshold value of 1.0, although traits such as SDM, TDM, and LDM did exhibit values close to 1.0 [23]. The discrepancy between this and the previously published experiments may have been related to the origin of the genetic material. While the hybrids had been developed through controlled crosses, and thus exhibited directed genetic variability, the ecotypes evaluated here were collected under natural conditions, subject to broad environmental variation. Therefore, although genetic diversity may be high among ecotypes, increased residual variance caused by environmental factors can reduce CV_r_, masking the expression of genetic potential. Nevertheless, as emphasized by Resende and Duarte [45], satisfactory levels of selection accuracy can still be achieved through an appropriate number of replications and adequate experimental precision. This is a crucial aspect in defining effective selection strategies for *P. nicorae*.

The decomposition of variances revealed that genetic variance ($\sigma_{g}^{2}$) was significant for most traits and highlighted the presence of genetic variability among the evaluated genotypes (Figure 1b). The highest values of $\sigma_{g}^{2}$ were observed for growth habit (GH, 45.38%), number of tillers (NT, 15.53%), and cold tolerance (CT, 10.04%). These values suggest a greater potential for selection response for these traits. In contrast, HI and FP had the lowest values ($\sigma_{g}^{2}$= 0.24 and $\sigma_{g}^{2}$ = 0.37, respectively), indicating a greater influence of non-genetic factors on their phenotypic expression (Figure 1a). The accurate estimation of variance components is essential for identifying superior genotypes, predicting genetic values, and determining expected selection gains [46]. The results indicated that NT and GH present the highest predominance of genotypic variance over environmental variance (${\hat{\sigma }}_{e}^{2}$), suggesting that these traits are largely controlled by genetic factors (Figure 1a). For most traits, the permanent environmental variance was lower than the genotypic variance, except for the PH, FP, CT, HI, and LDM traits. Conversely, the variance due to the genotype × measurement interaction exceeded the genotypic variance for most traits, with the exception of GH. These findings confirm that NT and GH exhibit the greatest genetic contribution, which is crucial for more efficient genetic selection strategies.

Environmental variance ($\sigma_{e}^{2}$) was particularly high for FP (79.08), SDM (49.27), and IDM (46.05), reinforcing the need for tighter experimental control in order to evaluate these traits. High residual variance coefficients may be associated with experimental challenges, such as operational difficulties during harvesting, which can reduce the precision of estimates [47]. In addition, the genotype-by-measurement interaction variance ($\sigma_{gm}^{2}$) was significant for all traits. This provided evidence of the influence of environmental variation on phenotypic expression and underscores the importance of multi-environment evaluations in selecting superior *P. nicorae* genotypes. Similar results were reported by Silveira et al. [23] for *P. notatum*, in which traits such as LDM (39.1%), TDM (36.6%), SDM (32.6%), and IDM (32.3%) showed the highest proportions of genotype-by-measurement interaction variance. This supports the suggestion of a strong environmental influence on the expression of these traits. Additionally, the same authors reported that PH showed the highest relative genetic variance (47.7%), followed by SDM (26.8%), GH (25.4%), TDM (24.6%), and LDM (21.8%), confirming the presence of substantial genetic variability in these traits.

In the present study, most traits in *P. nicorae* exhibited moderate-to-high broad-sense heritability estimates among genotypic means (h^2^_mg_), with values ranging from 0.028 (FP) to 0.827 (GH). Traits such as GH (0.827), NT (0.716), FM (0.620), and TDM (0.574) stood out for their high heritability, indicating strong genetic control and favorable prospects for genetic gain. Conversely, traits like FP (0.028), HI (0.033), and CT (0.400) presented low-to-moderate heritability estimates, suggesting a greater influence of environmental variation or limited genotypic differentiation under the experimental conditions. Notably, GH and NT are key traits for forage systems. A prostrate or semi-prostrate growth habit (GH scores of 3–4) is associated with greater grazing tolerance and persistence under defoliation. Likewise, a higher number of tillers supports rapid canopy coverage, competitiveness against weeds, and potential resilience to abiotic stressors. Thus, the high genetic variation observed for these traits highlights their importance for the development of more persistent and adaptive forage cultivars in subtropical environments.

These findings reinforce the importance of heritability as a key parameter in breeding programs as it quantifies the proportion of phenotypic variance attributable to genetic factors [48]. In P. notatum, contrasting results have been reported depending on the genetic structure of the populations. Silveira et al. [22] observed heritability estimates close to 1.0 for all traits in hybrid populations while Silveira et al. [23] and Marcon et al. [21] reported more variable patterns across traits and populations. These discrepancies are likely associated with differences in genetic structure as controlled crosses tend to yield higher genotypic variance than ecotypes sampled under natural conditions, as in the present study.

Beyond the environmental influence, such discrepancies among studies may be attributed to differences in the genetic base of the populations under evaluation, their stage in the breeding process, and the experimental design adopted [49]. The characterization of genetic variance components in inbred populations is fundamental for evaluating selection efficiency and predicting genetic progress across selection cycles [50]. In our study with *P. nicorae*, although several traits exhibited considerable genetic variance, others showed strong environmental influence (Figure 1a), which limited the selection response potential, particularly for traits with low heritability.

In the present study, although genetic variance was greater for several traits, some exhibited stronger environmental influence, reinforcing the importance of selection strategies that account for both genetic effects and G × E interactions when recommending promising genotypes for forage improvement programs for *P. nicorae*. Despite this, the application of mixed models (REML/BLUP) enables the accurate estimation of genotypic values, even for traits with low heritability, and allows for the individual selection of superior genotypes [51]. The combination of substantial genetic variability and high heritability forms a solid foundation for genetic progress in breeding programs, especially when the goal is to recombine contrasting populations [52].

The predicted genetic values (u + g + gem) obtained via BLUP, along with selection gains (SG%) and the population mean (µ), enabled all 84 *P. nicorae* ecotypes to be ranked from most to least promising for the 10 traits evaluated (Appendix A). In general, broad variability was observed in genotypic mean values, reflecting the potential for selection gains in different morphophysiological and productive traits. For NT, a key trait in forage grasses, genotypic values ranged from 109.66 (N7.15) to 375.99 (N3.10) tillers per plant. Ecotype L3.10 exhibited the highest genotypic value (375.99) for tillering capacity, which was 81% more than the population mean (µ = 207.96), with an estimated selection gain of 58.45% (Appendix A). In comparison, Silveira et al. [22] reported a maximum value of 248.06 tillers plant^−1^ for hybrid 137 in a population of 84 *P. notatum* genotypes, with a genetic gain of 145.86. More recently, Silveira et al. [23] observed a maximum of 312.17 tillers plant^−1^ for hybrid D23 in a population of 195 genotypes, with a genetic gain of 102.22 and SG of 119.85%. Although SG values were higher in *P. notatum*, the average genotypic value observed in *P. nicorae* was higher, demonstrating the species’s plasticity even under less intensive selection.

Traits associated with biomass production also exhibited considerable variation among ecotypes. For FM, genotypic values ranged from 85.49 (N4.17) to 244.15 g plant^−1^ (N3.10), with the highest yield associated with a selection gain of 37.36%. A similar pattern was observed for aerial biomass components, such as LDM, SDM, and IDM, where N3.10 consistently outperformed the other ecotypes. This indicates that N3.10 has productive stability (Appendix A). For LDM, N3.10 showed a genotypic value of 96.00 g plant^−1^ (SG: 29.96%). In comparison, Silveira et al. [22] reported a value of 347.14 g plant^−1^ for hybrid 336 of *P. notatum* while Silveira et al. [23] highlighted hybrid D3 with 208.63 g plant^−1^ and a selection gain of 124.19%. Although *P. notatum* hybrids showed higher absolute productivity, the results for *P. nicorae* are promising, especially considering the natural, unimproved origin of the evaluated population.

Moreover, *P. nicorae* presents unique adaptive traits that make it a compelling alternative or complement to *P. notatum* in specific contexts. Field observations and recent studies [14] have shown that *P. nicorae* performs better on sandy and loamy soils, establishes rapidly in cooler subtropical uplands, and tolerates both drought and cold. These features are particularly relevant for environments where *P. notatum* underperforms. As a species with minimal breeding history, *P. nicorae* also retains broad genetic diversity, an asset for future selection aimed at improving forage productivity, persistence, and seed yield. Thus, the development of improved cultivars based on this species could expand the portfolio of forage options available to producers and fill ecological niches not yet addressed by current commercial grasses. Together, these findings underscore the substantial potential of *P. nicorae* for genetic improvement via recurrent selection.

Total dry matter (TDM), a key forage productivity indicator as it represents the nutritional fraction of the feed disregarding water content, showed wide variation among *P. nicorae* ecotypes, ranging from 45.32 to 125.44 g plant^−1^ (Appendix A). Once again, N3.10 had the highest genotypic value and a selection gain (41.84%). This continued to support its status as the most productive ecotype in the population. In comparison, Silveira et al. [22] reported TDM values up to 581.97 g plant^−1^ in *P. notatum* hybrids, and Silveira et al. [23] observed a maximum of 193.37 g plant^−1^ and selection gains up to 105.34 g plant^−1^. Although *P. notatum* hybrids performed better in absolute terms, likely a result of their advanced breeding background, the relative gains observed in *P. nicorae* reinforce the species’s improvement potential. The performance of ecotypes like N3.10 demonstrated that, even in natural populations, superior biomass accumulation could be achieved through few cycles of recurrent selection.

Conversely, traits such as LSR and CT showed ranked different ecotypes in the first position. Ecotype N4.14 had the highest genotypic value for LSR (4.48), with a selection gain of 29.82%, while N1.09 ranked first for CT (4.39; SG: 27.14%). These findings indicate that different *P. nicorae* genotypes may be targeted for specific breeding goals, such as improving forage quality—through higher leaf blade proportions—or enhancing adaptation to cold-prone environments (Appendix A). In *P. notatum* hybrid populations, Silveira et al. [22] reported a maximum LSR of 7.13 for hybrid Q4188, highlighting favorable morphological performance. In the study by Silveira et al. [23], hybrid A24 showed an even higher genotypic value (37.32) and an individual selection gain (ISG) of 254.57%, reflecting intensive and directed selection. Although *P. nicorae* values were lower in absolute terms, the identification of naturally superior ecotypes reinforces the species’s potential as a genetic resource for breeding programs, particularly in regions where resilience and environmental adaptation are key priorities.

Plant height (PH), conversely, exhibited the lowest genotypic variability among the traits evaluated. Ecotype N2.02 had the highest genotypic value (65.46 cm), with a modest selection gain (SG: 5.21%), which indicated that there was limited genetic amplitude for this trait. However, this does not necessarily restrict the species’s use. Specifically, *P. nicorae* naturally has a shorter stature compared to other species in the genus. A shorter PH is a morphological trait associated with a tufted, prostrate growth habit, which favors denser canopy formation and improved grazing tolerance and better ground cover for soil conservation. Still, the genotypic values observed in *P. nicorae* were significantly higher than those recorded in *P. notatum* hybrid populations. Silveira et al. [22] showed that hybrid 437 reached 31.20 cm while Silveira et al. [23] reported a maximum of 23.07 cm for hybrid D17. These contrasts reveal not only morphological differences between species but also distinct ecological strategies and potential uses.

Overall, the results of this study highlight the superiority of ecotypes such as N3.10, N1.05, and N2.06, which produced the highest genotypic values and selection gains but also showed phenotypic stability across evaluated environments. These genotypes represent strategic materials to advance selection cycles and develop high-performance, broadly adapted cultivars. The comparison with *P. notatum* hybrids emphasizes that *P. nicorae* harbors valuable, yet underutilized, genetic resources with significant potential to contribute to the improvement of forage crops adapted to subtropical environments and sustainable management systems.

Superiority in multiple traits of agronomic interest is a key criterion for the successful selection of new forage genotypes [53]. In this context, the use of multivariate selection indices has proven to be an effective approach to address the complexity of simultaneous selection for agronomic, morphological, and adaptive traits [54]. The concept of the ideotype, originally proposed by Donald [55], underpins this approach by defining the ideal genotype as one that combines, in a balanced way, desirable attributes for specific production goals and environments. Based on this concept, Olivoto and Nardino [37] proposed the MGIDI index, which quantifies the multivariate distance between each evaluated genotype and a defined ideotype.

In the present study, the ideotype was constructed according to the main goals of forage grass breeding programs, prioritizing genotypes with high values for NT, FM, LDM, TDM, LSR, CT, GH, and PH, and simultaneously, low values for SDM and IDM. The harvest index (HI) and FP traits were excluded from ideotype construction due to the absence of significant genetic variability. The application of the MGIDI index enabled the ranking of the 84 *P. nicorae* ecotypes based on their distance to the ideotype, resulting in the selection of the 17 most promising genotypes, or 20% of the total ecotypes, under a 15% selection pressure (Figure 2). Ecotypes N3.10, N3.17, N2.06, N4.09, N3.18, N3.09, N2.07, N1.21, N3.04, N8.04, N5.01, N1.06, N3.05, N6.22, N1.18, N2.11, and N4.08 showed the shortest distance to the ideotype. The result indicated these ecotypes exhibited a balanced combination of traits related to productivity, morphological quality, and adaptability.

Although individual genetic values per trait are detailed in Appendix A, most of the genotypes selected via MGIDI were also identified as top performers in the univariate models. The results reported here demonstrated there was consistency among the selection methods. The use of MGIDI as a decision-support tool provides a more integrated and accurate selection strategy, which reduces subjectivity in genotype ranking and maximizes the likelihood of sustainable genetic progress. This strategy is particularly valuable for species such as *P. nicorae*, which is still undergoing domestication, as it enables the identification of superior ecotypes even within genetically broad, unimproved populations.

## 5. Conclusions

Considerable genetic variability was observed among *P. nicorae* ecotypes for the 12 evaluated traits, particularly for total dry matter, the number of tillers, fresh matter, and leaf dry matter, which exhibited high heritability and selection gain potential.

Ecotype N3.10 consistently had superior performance for biomass-related traits. It exhibited the highest predicted genotypic values and most substantial selection gains for multiple traits and is therefore considered a reference genotype for breeding programs targeting this species.

Traits associated with forage quality showed superiority in different ecotypes. Specifically, among the 84 ecotypes evaluated, N4.14 had the best leaf/stem ratio and N1.09 had the best level of cold tolerance. This will allow targeted selection to meet the specific goals of each breeding program.

The application of the MGIDI index enabled the identification of 17 ecotypes with balanced multivariate performance, characterized by shorter distances from the theoretical ideotype. The method demonstrates the effectiveness of simultaneous selection for multiple traits.

Comparison with published data from *P. notatum* hybrids revealed that *P. nicorae*, despite its lower level of domestication, includes agronomically competitive genotypes with high potential for use in forage production systems.

The integration of univariate analyses, genotypic stability estimates, and multivariate ideotype-based selection contributed to a more robust identification of superior materials, reaffirming the strategic value of *P. nicorae* as a promising genetic resource for forage breeding programs in subtropical regions.

## Figures and Tables

**Table 1:** Average monthly minimum (min) and maximum (max) air temperature (°C) and total monthly rainfall (mm) during the experimental period (November 2020–April 2023) with the 50 yr average (1969–2019).

Months	Temperature (°C, Min–Max)					Rainfall (mm)				
	2020	2021	2022	2023	54 yr avg.	2020	2021	2022	2023	54 yr avg.
Jan		20.3–30.0	20.2–33.3	25.8–33.0	19.3–30.2		67.8	110.1	31.2	109
Feb		18.8–29.7	19.0–30.7	24.0–30.6	19.0–29.4		31.2	77.2	67.8	100
Mar		18.9–28.7	17.6–27.7	25.0–31.5	18.0–28.2		86.4	98.1	5.8	83
Apr		15.4–27.4	14.1–24.3		14.5–25.2		5.8	118.2		74
May		8.4–20.8	10.9–19.7		11.2–21.8		147.6	94.4		85
Jun		9.3–18.6	7.9–17.3		8.7–18.6		120.8	97.6		109
Jul		6.7–19.2	11.1–21.1		8.5–18.7		52.6	131.4		145
Aug		11.0–21.5	9.2–20.1		9.7–20.2		121.4	92.2		115
Sep		13.7–22.4	10.7–21.6		11.2–21.4		176.4	58.6		159
Oct	15.5–25.0	13.5–25.4	13.4–24.1		13.8–24.0		28.8	44.4		128
Nov	16.7–27.3	15.9–27.6	14.7–27.1		15.4–26.6	109.6	56.4	49.8		107
Dec	17.2–32.0	17.9–29.1	17.1–31.9		17.5–29.0	44.2	42.8	53.6		94

**Table 2:** Soil chemical attributes (0–0.2 m) during the experimental period (2020–2023).

Variable	Units	2020	2021	2022
Clay	(%)	17	22	29
pH	(H_2_O)	4.4	4.6	4.2
SMP index	-	6	5.7	5.5
O.M.	(%)	1.2	1.6	1.1
P	mg/dm^3^	6.6	9.5	11
K	mg/dm^3^	51	64	87
Al	cmol_c_/dm^3^	1.1	0.8	1.1
Ca	cmol_c_/dm^3^	0.6	1.1	0.3
Mg	cmol_c_/dm^3^	0.2	0.3	0.1
H + Al	cmol_c_/dm^3^	4.4	6.2	7.7
CTC_effective_	cmol_c_/dm^3^	5.36	7.78	8.4
CTC_pH7_	cmol_c_/dm^3^	17	19	7
V	%	17	19	7
M	%	53	33	66
S	mg/dm^3^	5.6	11	18
Cu	mg/dm^3^	1	1.1	1.2
Zn	mg/dm^3^	1	1.3	1
Mn	mg/dm^3^	18	67	43
B	mg/dm^3^	0.3	0.2	0.4

Notes: O.M.: organic matter; P: phosphorus; K: potassium; Al: aluminum; Ca: calcium; Mg: magnesium; H + Al: potential acidity; CEC_effective_: cation exchange capacity determined by soil pH; CTC_pH7_: estimated cation exchange capacity at pH 7; V: base saturation; m: aluminum saturation; S: sulfur; Cu: copper; Zn: zinc; Mn: manganese; B: boron; -: dimensionless.

**Table 3:** Likelihood values (LRT) of deviance analysis (ANADEV) and experimental parameters for the 12 forage traits measured on 84 ecotypes of *P. nicorae* Parodi.

Effects	NT (Tillers Plant^−1^)	FM (g Plant^−1^)	LDM (g Plant^−1^)	SDM (g Plant^−1^)	IDM (g Plant^−1^)	TDM (g Plant^−1^)	LSR	HI	CT	FP	**GH**	**PH** **(cm)**
Full model	36,404.77	37,909.20	34,132.21	16,407.65	12,348.90	33,497.38	6635.83	−11,662.29	801.24	−1197.18	90.26	5486.26
Genotype	36,489.74	37,956.31	34,146.62	16,418.08	12,364.09	33,532.62	6661.55	−11,662.22	809.63	−1197.25	211.52	5497.80
Environment	36,432.95	37,952.24	34,234.11	16,426.91	12,349.53	33,561.22	6648.39	−11,264.48	834.62	−1193.77	164.35	5511.13
Interaction _GxA_	42,801.97	40,979.57	35,216.43	17,486.52	13,648.72	35,223.72	8718.61	−11,204.75	858.19	−1171.75	138.45	5544.04
LRT_g_ (X^2^)	84.97 **	47.11 **	14.41 **	10.43 **	15.19 **	35.24 **	25.72 **	0.07 ^ns^	8.39 **	−0.07 ^ns^	121.26 **	11.54 **
LRT_e_(X^2^)	28.18 **	43.04 **	101.90 **	19.26 **	0.63 ^ns^	63.84 **	12.56 **	397.81 **	33.38 **	3.41 ^ns^	74.09 **	24.87 **
LRT_GxE_(X^2^)	6397.20 **	3070.37 **	1084.22 **	1078.87 **	1299.82 **	1726.34 **	2082.78 **	457.54 **	56.95 **	25.43 **	48.19 **	57.78 **
µ	207.96	155.18	62.32	6.55	3.86	75.89	2.93	0.82	3.03	0.86	2.87	57.02
CV_e_ (%)	11.73	21.71	39.43	49.13	51.45	28.73	32.80	15.65	23.32	34.02	15.76	13.32
CV_g_ (%)	17.51	14.39	11.81	12.33	14.58	13.07	13.99	0.99	10.00	2.34	19.37	3.44
VI_e_ (%)	5.87	10.85	19.71	24.57	25.73	14.36	16.40	7.82	11.66	17.01	7.88	6.66
VI_g_ (%)	8.76	7.19	5.91	6.17	7.29	6.54	7.00	0.50	5.00	1.17	9.69	1.72
CV_r_	1.49	0.66	0.30	0.25	0.28	0.45	0.43	0.06	0.43	0.07	1.23	0.26

**: significant according to the likelihood ratio test at 1% (LRT, 1% = 6.63) probability levels, using a chi-square (χ^2^) distribution with 1 degree of freedom; ns: not significant. µ: grand mean; CV_e_ (%): experimental coefficient of variation; CV_g_ (%): genetic coefficient of variation; IV_e_ (%): experimental variation index; IV_g_ (%): genetic variation index; CV_r_: relative coefficient of variation (CV_e_/CV_g_). NT: number of tillers; FM: fresh matter; LDM: leaf dry matter; SDM: stem dry matter; IDM: inflorescence dry matter; TDM: total dry matter; LSR: leaf/stem ratio; HI: harvest index; CT: cold tolerance; FP: forage persistence; GH: growth habit and PH: plant height.

## Data Availability

The data presented in this study are available on request from the corresponding author. The data are not publicly available due to ongoing analyses and project restrictions.

## Abbreviations

The following abbreviations are used in this manuscript:

UFRGS	Universidade Federal do Rio Grande do Sul
DPFA	Department of Forage Plants and Agrometeorology
Cfa	Humid subtropical climate, no dry season, hot summer
LD	Linear dichroism
RCBD	Randomized complete block design
OM	Organic matter
P	Phosphorus
K	Potassium
Al	Aluminum
Ca	Calcium
Mg	magnesium
H + Al	Potential acidity
CEC_effective_	Cation exchange capacity determined by soil pH
CTC_pH7_	Estimated cation exchange capacity at pH 7
V	Base saturation
m	Aluminum saturation
S	Sulfur
Cu	Copper
Zn	Zinc
Mn	Manganese
B	Boron
NT	Number of tillers
FM	Fresh mass
LDM	Leaf dry mass
SDM	Stem dry mass
IDM	Inflorescence dry mass
TDM	Total dry mass
LSR	Leaf-to-stem ratio
HI	Harvest index
CT	Cold tolerance
FP	Forage persistence
GH	Growth habit
PH	Plant height
ANADEV	Analysis of Deviance
BLUP	Best linear unbiased prediction
CV_e_ (%)	Experimental coefficient of variation
CV_g_ (%)	Genetic coefficient of variation
CV_r_	Relative coefficient of variation (CV_e_/CV_g_)
HMRPGV	Harmonic mean of relative performance of genotypic values
$\sigma_{e}^{2}$	Environmental variance
$\sigma_{gm}^{2}$	Genotype-by-measurement interaction variance
$\sigma_{perm}^{2}$	Permanent environment variance
$\sigma_{g}^{2}$	Genotypic variance
h^2^	Broad-sense heritability of individual plots, considering total genotypic effects; ρ: repeatability at the plot level
$C_{perm}^{2}$	Coefficient of determination of plot effects
$C_{gm}^{2}$	Coefficient for determining the effects of the genotypes x measurements interaction
$r_{gmed}$	Genotypic correlation through measurements
$h_{\mathrm{mg}}^{2}$	Heritability of the genotype mean
u + g + gem	Average genotypic values
SG%	Selection gains
IV_e_ (%)	Experimental variation index
IV_g_ (%)	Genetic variation index
µ	Grand mean

## Appendix A

**Table A1:** Predicted genetic gain estimates (BLUP) for 10 forage traits in 84 *P. nicorae* Parodi ecotypes based on mean performance across experimental years.

					**NT (Tillers Plant^−1^)**					**FM (** **g Plant^−1^)**				
**Order**	**Ecotype**	**g**	**u + g**	**Gain**	**µ_n_**	**u + g + gem**	**SG**	**Ecotypes**	**g**	**u + g**	**Gain**	**µ_n_**	**u + g + gem**	**SG**
1	N3.10	121.55	329.51	121.55	329.51	375.99	58.45	N3.10	57.97	213.14	57.97	213.14	244.15	37.36
2	N3.17	89.28	297.24	105.42	313.38	331.38	42.93	N2.06	35.85	191.02	46.91	202.08	210.20	23.10
3	N3.12	52.98	260.94	87.94	295.90	281.20	25.48	N3.17	32.32	187.50	42.04	197.22	204.78	20.83
4	N5.09	48.87	256.83	78.17	286.13	275.51	23.50	N4.09	30.78	185.96	39.23	194.41	202.43	19.84
5	N2.11	42.08	250.04	70.95	278.91	266.13	20.24	N5.01	29.35	184.53	37.25	192.43	200.23	18.92
6	N2.07	36.90	244.86	65.28	273.24	258.97	17.74	N1.21	28.48	183.65	35.79	190.97	198.89	18.35
7	N8.04	36.60	244.56	61.18	269.14	258.55	17.60	N2.07	22.87	178.04	33.94	189.12	190.27	14.74
8	N4.09	36.52	244.48	58.10	266.06	258.44	17.56	N2.11	21.87	177.05	32.44	187.61	188.74	14.09
9	N5.01	35.02	242.98	55.53	263.49	256.38	16.84	N1.05	21.37	176.55	31.21	186.38	187.98	13.77
10	N3.18	33.27	241.23	53.31	261.27	253.95	16.00	N3.09	21.37	176.55	30.22	185.40	187.98	13.77
11	N3.09	33.10	241.06	51.47	259.43	253.72	15.92	N4.08	21.25	176.43	29.41	184.58	187.80	13.70
12	N3.03	30.65	238.61	49.74	257.70	250.33	14.74	N1.06	18.46	173.64	28.50	183.67	183.51	11.90
13	N5.03	30.37	238.33	48.25	256.21	249.95	14.61	N5.12	17.43	172.61	27.64	182.82	181.93	11.23
14	N3.16	29.64	237.60	46.92	254.88	248.94	14.25	N3.12	16.33	171.51	26.84	182.01	180.24	10.52
15	N2.06	28.65	236.61	45.70	253.66	247.57	13.78	N3.18	16.25	171.42	26.13	181.31	180.11	10.47
16	N1.21	28.17	236.13	44.60	252.56	246.90	13.55	N2.16	15.40	170.58	25.46	180.64	178.82	9.93
17	N2.16	25.01	232.97	43.45	251.41	242.53	12.02	N3.16	15.24	170.42	24.86	180.04	178.57	9.82
18	N5.20	22.07	230.03	42.26	250.22	238.47	10.61	N1.19	15.24	170.42	24.32	179.50	178.57	9.82
19	N3.05	21.56	229.52	41.17	249.13	237.76	10.37	N3.05	11.58	166.76	23.65	178.83	172.95	7.46
20	N4.15	20.29	228.25	40.13	248.09	236.01	9.76	N3.04	11.09	166.26	23.03	178.20	172.19	7.15
21	N4.08	19.59	227.55	39.15	247.11	235.04	9.42	N2.02	10.12	165.29	22.41	177.59	170.70	6.52
22	N4.07	19.48	227.44	38.26	246.22	234.88	9.37	N5.09	9.14	164.32	21.81	176.98	169.21	5.89
23	N5.18	16.73	224.69	37.32	245.28	231.09	8.05	N8.04	9.13	164.30	21.26	176.43	169.19	5.88
24	N3.04	16.64	224.60	36.46	244.42	230.96	8.00	N4.07	8.91	164.08	20.74	175.92	168.85	5.74
25	N6.12	15.65	223.61	35.63	243.59	229.59	7.52	N1.22	8.37	163.54	20.25	175.42	168.02	5.39
26	N2.22	14.35	222.31	34.81	242.77	227.79	6.90	N1.18	7.94	163.11	19.77	174.95	167.36	5.11
27	N1.10	12.84	220.80	34.00	241.95	225.72	6.18	N5.18	7.80	162.98	19.33	174.51	167.16	5.03
28	N1.05	12.58	220.54	33.23	241.19	225.34	6.05	N5.03	7.75	162.92	18.92	174.09	167.07	4.99
29	N4.13	11.53	219.49	32.48	240.44	223.89	5.54	N6.22	6.75	161.93	18.50	173.67	165.54	4.35
30	N4.20	11.18	219.14	31.77	239.73	223.42	5.38	N4.06	6.72	161.89	18.10	173.28	165.49	4.33
31	N5.12	7.95	215.91	31.00	238.96	218.94	3.82	N5.20	6.36	161.54	17.73	172.90	164.94	4.10
32	N1.18	7.18	215.14	30.26	238.22	217.89	3.45	N5.19	6.26	161.43	17.37	172.54	164.78	4.03
33	N2.10	6.99	214.95	29.55	237.51	217.62	3.36	N3.15	6.01	161.18	17.02	172.20	164.40	3.87
34	N5.22	6.88	214.84	28.89	236.85	217.47	3.31	N3.19	5.38	160.56	16.68	171.86	163.44	3.47
35	N5.19	6.59	214.55	28.25	236.21	217.07	3.17	N6.08	4.22	159.40	16.32	171.50	161.66	2.72
36	N2.02	4.91	212.87	27.60	235.56	214.75	2.36	N6.12	3.89	159.07	15.98	171.16	161.15	2.51
37	N4.05	3.52	211.48	26.95	234.91	212.82	1.69	N3.03	3.70	158.88	15.65	170.82	160.86	2.38
38	N4.06	1.32	209.28	26.28	234.24	209.78	0.63	N2.10	2.59	157.77	15.30	170.48	159.15	1.67
39	N1.17	−0.36	207.60	25.59	233.55	207.46	−0.17	N3.02	2.11	157.29	14.97	170.14	158.42	1.36
40	N2.13	−1.30	206.66	24.92	232.88	206.17	−0.62	N2.13	1.29	156.46	14.62	169.80	157.15	0.83
41	N3.02	−3.90	204.06	24.22	232.18	202.57	−1.87	N6.19	−0.25	154.93	14.26	169.44	154.79	−0.16
42	N7.20	−4.10	203.86	23.54	231.50	202.29	−1.97	N1.17	−0.57	154.61	13.91	169.08	154.31	−0.37
43	N1.06	−4.16	203.80	22.90	230.86	202.21	−2.00	N5.15	−1.03	154.14	13.56	168.74	153.59	−0.67
44	N5.05	−4.23	203.73	22.28	230.24	202.11	−2.04	N6.05	−1.67	153.51	13.21	168.39	152.61	−1.08
45	N6.01	−5.11	202.85	21.67	229.63	200.89	−2.46	N4.21	−1.70	153.47	12.88	168.06	152.56	−1.10
46	N3.15	−5.66	202.30	21.08	229.04	200.14	−2.72	N4.13	−1.90	153.28	12.56	167.74	152.26	−1.22
47	N2.17	−5.80	202.16	20.51	228.47	199.94	−2.79	N5.21	−3.60	151.57	12.22	167.39	149.65	−2.32
48	N5.15	−7.02	200.94	19.93	227.89	198.26	−3.37	N5.22	−3.94	151.24	11.88	167.06	149.13	−2.54
49	N3.22	−7.10	200.86	19.38	227.34	198.15	−3.41	N4.05	−4.42	150.76	11.55	166.72	148.39	−2.85
50	N5.16	−9.04	198.92	18.81	226.77	195.47	−4.35	N4.15	−4.53	150.65	11.23	166.40	148.22	−2.92
51	N5.21	−9.28	198.68	18.26	226.22	195.13	−4.46	N2.01	−5.88	149.30	10.89	166.07	146.16	−3.79
52	N6.08	−10.00	197.96	17.72	225.68	194.14	−4.81	N2.22	−6.90	148.28	10.55	165.73	144.59	−4.44
53	N3.07	−10.75	197.21	17.18	225.14	193.09	−5.17	N1.10	−7.58	147.60	10.21	165.38	143.55	−4.88
54	N2.01	−14.48	193.48	16.60	224.56	187.94	−6.96	N3.08	−7.89	147.28	9.87	165.05	143.06	−5.09
55	N3.06	−14.92	193.04	16.02	223.98	187.33	−7.18	N5.05	−8.20	146.98	9.54	164.72	142.59	−5.28
56	N6.22	−15.32	192.64	15.46	223.42	186.78	−7.37	N5.16	−8.25	146.93	9.23	164.40	142.52	−5.32
57	N1.22	−16.60	191.36	14.90	222.86	185.02	−7.98	N6.18	−8.47	146.71	8.92	164.09	142.18	−5.46
58	N6.19	−17.94	190.02	14.33	222.29	183.16	−8.63	N3.07	−9.22	145.96	8.60	163.78	141.03	−5.94
59	N3.19	−19.29	188.67	13.76	221.72	181.29	−9.28	N4.20	−9.25	145.93	8.30	163.48	140.98	−5.96
60	N4.10	−19.96	188.00	13.20	221.16	180.36	−9.60	N6.01	−9.84	145.34	8.00	163.17	140.07	−6.34
61	N4.21	−20.80	187.16	12.65	220.60	179.21	−10.00	N5.10	−9.95	145.23	7.70	162.88	139.91	−6.41
62	N1.09	−21.64	186.32	12.09	220.05	178.05	−10.41	N3.22	−11.29	143.89	7.40	162.57	137.85	−7.28
63	N1.07	−22.79	185.17	11.54	219.50	176.45	−10.96	N1.09	−11.39	143.78	7.10	162.28	137.69	−7.34
64	N5.02	−22.97	184.99	11.00	218.96	176.21	−11.05	N5.02	−12.61	142.57	6.79	161.97	135.82	−8.13
65	N4.14	−23.51	184.45	10.47	218.43	175.46	−11.31	N5.14	−12.79	142.39	6.49	161.67	135.55	−8.24
66	N5.10	−24.13	183.83	9.94	217.90	174.61	−11.60	N2.17	−14.33	140.84	6.17	161.35	133.18	−9.24
67	N1.19	−24.65	183.31	9.43	217.39	173.88	−11.85	N4.14	−14.67	140.51	5.86	161.04	132.66	−9.45
68	N5.11	−25.43	182.53	8.92	216.87	172.81	−12.23	N4.10	−15.06	140.11	5.56	160.73	132.06	−9.71
69	N2.18	−25.69	182.27	8.41	216.37	172.45	−12.35	N7.20	−15.18	139.99	5.25	160.43	131.87	−9.78
70	N1.13	−26.63	181.33	7.91	215.87	171.14	−12.81	N8.03	−15.23	139.94	4.96	160.14	131.79	−9.82
71	N5.06	−29.14	178.82	7.39	215.35	167.67	−14.01	N5.06	−15.26	139.92	4.68	159.85	131.76	−9.83
72	N4.19	−29.17	178.79	6.88	214.84	167.63	−14.03	N2.18	−17.83	137.34	4.36	159.54	127.80	−11.49
73	N8.03	−30.05	177.91	6.38	214.34	166.42	−14.45	N1.16	−18.10	137.08	4.06	159.23	127.40	−11.66
74	N4.12	−30.42	177.54	5.88	213.84	165.91	−14.63	N4.19	−19.80	135.37	3.73	158.91	124.78	−12.76
75	N3.08	−30.48	177.48	5.40	213.35	165.83	−14.66	N1.12	−19.85	135.32	3.42	158.60	124.70	−12.79
76	N1.16	−31.75	176.21	4.91	212.87	164.07	−15.27	N3.06	−20.02	135.16	3.11	158.29	124.45	−12.90
77	N1.15	−32.22	175.74	4.42	212.38	163.42	−15.49	N1.14	−22.86	132.32	2.77	157.95	120.09	−14.73
78	N5.14	−32.27	175.69	3.95	211.91	163.36	−15.52	N1.07	−25.00	130.18	2.42	157.60	116.81	−16.11
79	N6.05	−32.54	175.42	3.49	211.45	162.97	−15.65	N5.11	−25.01	130.17	2.07	157.25	116.80	−16.11
80	N1.12	−46.03	161.93	2.87	210.83	144.33	−22.13	N1.13	−25.72	129.46	1.72	156.90	115.70	−16.57
81	N1.14	−47.95	160.01	2.25	210.21	141.68	−23.06	N7.15	−28.29	126.89	1.35	156.53	111.76	−18.23
82	N6.18	−48.90	159.06	1.62	209.58	140.36	−23.51	N4.12	−31.70	123.47	0.95	156.13	106.51	−20.43
83	N4.17	−61.89	146.07	0.86	208.82	122.41	−29.76	N1.15	−32.52	122.65	0.55	155.72	105.26	−20.96
84	N7.15	−71.11	136.85	0.00	207.96	109.66	−34.19	N4.17	−45.40	109.78	0.00	155.18	85.49	−29.26
					**LDM (g plant^−1^)**					**SDM (g plant^−1^)**				
**Order**	**Ecotypes**	**g**	**u + g**	**Gain**	**µ_n_**	**u + g + gem**	**SG**	**Ecotypes**	**g**	**u + g**	**Gain**	**µ_n_**	**u + g + gem**	**SG**
1	N3.10	18.67	80.98	18.67	80.98	96.00	29.96	N3.10	1.94	8.49	1.94	8.49	10.69	29.65
2	N2.06	10.46	72.77	14.56	76.88	81.19	16.78	N3.17	1.40	7.94	1.67	8.21	9.53	21.31
3	N1.05	9.67	71.99	12.93	75.25	79.77	15.52	N2.06	1.23	7.77	1.52	8.07	9.17	18.77
4	N3.17	8.57	70.88	11.84	74.16	77.77	13.75	N3.05	0.62	7.17	1.30	7.84	7.88	9.52
5	N2.11	7.20	69.51	10.91	73.23	75.30	11.55	N6.22	0.62	7.16	1.16	7.71	7.86	9.39
6	N5.01	6.46	68.77	10.17	72.49	73.97	10.37	N1.06	0.61	7.15	1.07	7.61	7.84	9.26
7	N3.12	6.18	68.50	9.60	71.92	73.47	9.92	N1.21	0.60	7.15	1.00	7.55	7.84	9.22
8	N3.18	6.05	68.37	9.16	71.47	73.24	9.71	N1.19	0.54	7.09	0.94	7.49	7.70	8.29
9	N8.04	5.63	67.94	8.76	71.08	72.47	9.03	N3.04	0.51	7.05	0.90	7.44	7.63	7.76
10	N1.06	5.56	67.87	8.44	70.76	72.34	8.92	N3.09	0.47	7.02	0.85	7.40	7.56	7.23
11	N3.03	5.29	67.60	8.16	70.47	71.85	8.48	N5.12	0.47	7.01	0.82	7.36	7.54	7.13
12	N4.09	4.80	67.12	7.88	70.19	70.98	7.71	N8.04	0.45	7.00	0.79	7.33	7.51	6.89
13	N6.22	4.46	66.78	7.61	69.93	70.37	7.16	N4.09	0.43	6.98	0.76	7.31	7.47	6.60
14	N5.15	4.31	66.62	7.38	69.69	70.09	6.91	N3.15	0.40	6.94	0.73	7.28	7.39	6.05
15	N3.08	4.27	66.58	7.17	69.49	70.02	6.85	N5.21	0.39	6.94	0.71	7.26	7.38	5.95
16	N4.08	4.07	66.39	6.98	69.29	69.66	6.53	N5.01	0.39	6.94	0.69	7.24	7.38	5.94
17	N5.14	3.99	66.31	6.80	69.12	69.52	6.41	N4.06	0.38	6.92	0.67	7.22	7.35	5.76
18	N3.09	3.62	65.93	6.63	68.94	68.84	5.80	N5.15	0.36	6.91	0.66	7.20	7.32	5.55
19	N1.21	3.42	65.74	6.46	68.77	68.49	5.49	N1.18	0.32	6.87	0.64	7.18	7.23	4.89
20	N2.07	3.38	65.70	6.30	68.62	68.42	5.43	N3.16	0.32	6.87	0.62	7.17	7.23	4.88
21	N1.18	3.12	65.44	6.15	68.47	67.95	5.01	N2.01	0.25	6.80	0.60	7.15	7.08	3.83
22	N3.15	2.41	64.73	5.98	68.30	66.66	3.87	N3.02	0.24	6.79	0.59	7.13	7.06	3.70
23	N3.04	2.29	64.61	5.82	68.14	66.46	3.68	N4.20	0.22	6.76	0.57	7.12	7.01	3.32
24	N2.02	2.16	64.47	5.67	67.98	66.21	3.46	N5.03	0.21	6.76	0.56	7.10	7.00	3.27
25	N5.12	2.13	64.44	5.53	67.84	66.16	3.42	N1.09	0.20	6.74	0.54	7.09	6.97	3.01
26	N8.03	1.93	64.24	5.39	67.70	65.80	3.09	N3.12	0.19	6.73	0.53	7.07	6.94	2.83
27	N1.22	1.87	64.19	5.26	67.57	65.70	3.01	N5.19	0.18	6.73	0.52	7.06	6.94	2.81
28	N4.15	1.79	64.10	5.13	67.45	65.54	2.87	N5.14	0.18	6.72	0.50	7.05	6.92	2.68
29	N4.06	1.77	64.08	5.02	67.33	65.50	2.84	N5.10	0.17	6.72	0.49	7.04	6.91	2.63
30	N3.02	1.28	63.59	4.89	67.21	64.62	2.05	N3.19	0.17	6.72	0.48	7.03	6.91	2.59
31	N2.16	1.19	63.50	4.77	67.09	64.46	1.91	N1.22	0.17	6.71	0.47	7.02	6.90	2.56
32	N1.19	1.12	63.43	4.66	66.97	64.33	1.79	N1.17	0.17	6.71	0.46	7.01	6.90	2.53
33	N1.09	1.04	63.35	4.55	66.87	64.19	1.66	N3.18	0.15	6.70	0.45	7.00	6.87	2.32
34	N6.12	0.99	63.31	4.45	66.76	64.10	1.59	N2.22	0.14	6.68	0.44	6.99	6.84	2.07
35	N5.21	0.92	63.23	4.34	66.66	63.97	1.47	N1.05	0.12	6.67	0.43	6.98	6.81	1.91
36	N5.05	0.86	63.17	4.25	66.56	63.87	1.38	N3.03	0.12	6.66	0.43	6.97	6.80	1.79
37	N4.21	0.65	62.96	4.15	66.47	63.48	1.04	N5.18	0.11	6.66	0.42	6.96	6.78	1.68
38	N3.05	0.57	62.89	4.06	66.37	63.35	0.92	N4.15	0.08	6.62	0.41	6.95	6.71	1.18
39	N3.19	0.57	62.88	3.97	66.28	63.34	0.91	N5.06	0.06	6.60	0.40	6.95	6.67	0.88
40	N4.07	0.15	62.47	3.87	66.19	62.59	0.24	N4.13	0.05	6.60	0.39	6.94	6.66	0.81
41	N6.08	−0.46	61.85	3.77	66.08	61.48	−0.74	N3.08	0.02	6.57	0.38	6.93	6.59	0.31
42	N3.16	−0.53	61.78	3.66	65.98	61.36	−0.85	N2.02	0.02	6.56	0.37	6.92	6.58	0.27
43	N2.13	−0.59	61.73	3.56	65.88	61.26	−0.94	N4.07	0.02	6.56	0.36	6.91	6.58	0.23
44	N4.20	−0.74	61.58	3.47	65.78	60.98	−1.19	N5.09	0.01	6.55	0.36	6.90	6.56	0.08
45	N5.03	−0.97	61.34	3.37	65.68	60.56	−1.56	N3.22	−0.02	6.53	0.35	6.89	6.51	−0.25
46	N2.01	−1.03	61.29	3.27	65.59	60.46	−1.65	N1.16	−0.02	6.53	0.34	6.89	6.50	−0.30
47	N5.18	−1.11	61.21	3.18	65.49	60.31	−1.78	N6.19	−0.04	6.50	0.33	6.88	6.46	−0.65
48	N1.12	−1.16	61.16	3.09	65.40	60.23	−1.85	N2.16	−0.06	6.49	0.32	6.87	6.42	−0.88
49	N5.16	−1.21	61.11	3.00	65.32	60.14	−1.93	N6.12	−0.06	6.48	0.32	6.86	6.41	−0.95
50	N5.20	−1.26	61.06	2.92	65.23	60.04	−2.02	N2.11	−0.08	6.47	0.31	6.85	6.38	−1.16
51	N4.13	−1.43	60.89	2.83	65.15	59.74	−2.29	N4.14	−0.09	6.46	0.30	6.85	6.35	−1.38
52	N2.10	−1.46	60.85	2.75	65.06	59.68	−2.35	N8.03	−0.09	6.45	0.29	6.84	6.35	−1.40
53	N1.17	−1.63	60.69	2.67	64.98	59.38	−2.61	N2.07	−0.11	6.43	0.28	6.83	6.31	−1.71
54	N4.05	−1.72	60.59	2.58	64.90	59.20	−2.77	N4.08	−0.12	6.43	0.28	6.82	6.29	−1.84
55	N6.05	−2.03	60.29	2.50	64.82	58.66	−3.25	N2.13	−0.13	6.41	0.27	6.82	6.26	−2.01
56	N1.07	−2.14	60.18	2.42	64.73	58.45	−3.43	N6.05	−0.17	6.37	0.26	6.81	6.18	−2.63
57	N1.16	−2.26	60.06	2.34	64.65	58.24	−3.63	N2.17	−0.17	6.37	0.25	6.80	6.17	−2.66
58	N4.19	−2.35	59.97	2.25	64.57	58.07	−3.77	N1.10	−0.18	6.36	0.25	6.79	6.15	−2.80
59	N2.22	−2.42	59.89	2.18	64.49	57.94	−3.89	N4.21	−0.24	6.31	0.24	6.78	6.04	−3.64
60	N5.09	−2.65	59.67	2.10	64.41	57.54	−4.25	N6.18	−0.24	6.31	0.23	6.78	6.03	−3.66
61	N5.19	−2.76	59.56	2.02	64.33	57.34	−4.43	N7.20	−0.26	6.29	0.22	6.77	5.99	−3.98
62	N6.18	−2.83	59.49	1.94	64.25	57.21	−4.54	N1.12	−0.26	6.29	0.21	6.76	5.99	−3.98
63	N5.10	−2.84	59.48	1.86	64.18	57.20	−4.55	N2.10	−0.31	6.23	0.21	6.75	5.88	−4.80
64	N7.20	−2.89	59.42	1.79	64.10	57.10	−4.64	N4.10	−0.34	6.21	0.20	6.74	5.83	−5.13
65	N1.14	−2.96	59.36	1.71	64.03	56.98	−4.74	N6.08	−0.35	6.19	0.19	6.74	5.79	−5.38
66	N5.22	−3.15	59.17	1.64	63.96	56.63	−5.05	N5.22	−0.40	6.15	0.18	6.73	5.69	−6.11
67	N6.19	−3.17	59.15	1.57	63.88	56.60	−5.09	N1.14	−0.45	6.10	0.17	6.72	5.59	−6.82
68	N1.10	−3.47	58.85	1.49	63.81	56.06	−5.56	N1.07	−0.47	6.07	0.16	6.71	5.54	−7.23
69	N5.11	−3.57	58.75	1.42	63.74	55.88	−5.73	N4.05	−0.47	6.07	0.15	6.70	5.53	−7.25
70	N4.10	−3.82	58.50	1.35	63.66	55.43	−6.13	N4.19	−0.53	6.01	0.14	6.69	5.41	−8.14
71	N2.17	−3.86	58.46	1.27	63.59	55.35	−6.19	N5.20	−0.57	5.98	0.13	6.68	5.34	−8.64
72	N5.02	−3.98	58.34	1.20	63.52	55.14	−6.38	N5.02	−0.58	5.97	0.12	6.67	5.31	−8.82
73	N3.07	−4.02	58.29	1.13	63.44	55.06	−6.46	N6.01	−0.60	5.95	0.11	6.66	5.27	−9.11
74	N4.14	−4.83	57.49	1.05	63.36	53.61	−7.75	N2.18	−0.61	5.94	0.10	6.65	5.25	−9.28
75	N6.01	−5.39	56.93	0.96	63.28	52.59	−8.65	N5.16	−0.62	5.93	0.09	6.64	5.22	−9.47
76	N3.22	−5.40	56.92	0.88	63.19	52.58	−8.66	N4.12	−0.67	5.87	0.08	6.63	5.11	−10.30
77	N3.06	−5.47	56.84	0.80	63.11	52.44	−8.78	N3.07	−0.68	5.86	0.07	6.62	5.08	−10.45
78	N5.06	−5.78	56.53	0.71	63.03	51.88	−9.28	N5.05	−0.69	5.86	0.06	6.61	5.08	−10.49
79	N1.15	−6.95	55.36	0.62	62.93	49.77	−11.16	N7.15	−0.72	5.82	0.05	6.60	5.00	−11.05
80	N1.13	−7.78	54.53	0.51	62.83	48.27	−12.49	N1.15	−0.73	5.82	0.04	6.59	4.99	−11.10
81	N7.15	−8.91	53.41	0.39	62.71	46.24	−14.30	N1.13	−0.73	5.82	0.03	6.58	4.99	−11.10
82	N4.12	−9.95	52.37	0.27	62.58	44.37	−15.96	N3.06	−0.77	5.77	0.02	6.57	4.90	−11.78
83	N2.18	−10.35	51.96	0.14	62.46	43.64	−16.61	N5.11	−0.84	5.70	0.01	6.56	4.74	−12.90
84	N4.17	−11.60	50.72	0.00	62.32	41.39	−18.61	N4.17	−1.19	5.36	0.00	6.55	4.01	−18.12
					**IDM (g plant^−1^)**					**TDM (g plant^−1^)**				
**Order**	**Ecotypes**	**g**	**u + g**	**Gain**	**µ_n_**	**u + g + gem**	**SG**	**Ecotypes**	**g**	**u + g**	**Gain**	**µ_n_**	**u + g + gem**	**SG**
1	N3.10	0.95	4.82	0.95	4.82	5.81	24.70	N3.10	31.75	107.65	31.75	107.65	125.44	41.84
2	N3.18	0.90	4.77	0.93	4.79	5.71	23.40	N2.06	15.25	91.14	23.50	99.40	99.69	20.09
3	N2.16	0.70	4.57	0.85	4.72	5.30	18.21	N3.17	12.64	88.54	19.88	95.78	95.62	16.66
4	N2.07	0.69	4.55	0.81	4.68	5.27	17.85	N1.05	10.77	86.66	17.60	93.50	92.70	14.19
5	N3.17	0.66	4.53	0.78	4.65	5.22	17.19	N1.21	10.30	86.20	16.14	92.04	91.97	13.58
6	N3.04	0.60	4.47	0.75	4.62	5.09	15.58	N4.09	9.92	85.81	15.11	91.00	91.37	13.07
7	N2.11	0.59	4.46	0.73	4.59	5.07	15.29	N3.18	9.64	85.53	14.33	90.22	90.93	12.70
8	N4.07	0.59	4.45	0.71	4.58	5.06	15.14	N1.06	8.68	84.57	13.62	89.51	89.43	11.43
9	N2.02	0.56	4.43	0.70	4.56	5.01	14.58	N5.01	8.35	84.24	13.03	88.93	88.92	11.00
10	N1.05	0.49	4.35	0.67	4.54	4.86	12.66	N6.22	8.25	84.14	12.56	88.45	88.77	10.87
11	N1.18	0.46	4.32	0.66	4.52	4.80	11.84	N3.09	7.81	83.71	12.12	88.02	88.08	10.29
12	N3.09	0.41	4.28	0.63	4.50	4.71	10.68	N8.04	7.29	83.19	11.72	87.62	87.27	9.61
13	N1.21	0.41	4.27	0.62	4.48	4.70	10.54	N2.11	7.28	83.18	11.38	87.27	87.26	9.60
14	N5.20	0.37	4.24	0.60	4.46	4.63	9.68	N5.12	7.12	83.02	11.08	86.97	87.01	9.38
15	N2.06	0.37	4.24	0.58	4.45	4.63	9.66	N2.07	6.94	82.83	10.80	86.69	86.72	9.14
16	N5.15	0.36	4.22	0.57	4.44	4.59	9.23	N4.06	6.62	82.51	10.54	86.43	86.22	8.72
17	N6.08	0.35	4.22	0.56	4.42	4.58	9.08	N1.18	6.00	81.89	10.27	86.17	85.26	7.91
18	N4.09	0.34	4.21	0.55	4.41	4.56	8.82	N3.04	5.81	81.70	10.02	85.92	84.96	7.66
19	N3.03	0.28	4.15	0.53	4.40	4.44	7.33	N3.12	5.28	81.17	9.77	85.67	84.12	6.95
20	N4.08	0.26	4.12	0.52	4.38	4.39	6.61	N4.08	4.80	80.69	9.53	85.42	83.38	6.32
21	N5.01	0.22	4.09	0.50	4.37	4.31	5.70	N3.05	4.54	80.44	9.29	85.18	82.98	5.99
22	N5.18	0.21	4.07	0.49	4.35	4.29	5.35	N3.03	4.33	80.22	9.06	84.96	82.64	5.70
23	N5.09	0.20	4.06	0.48	4.34	4.26	5.07	N2.02	4.08	79.97	8.85	84.74	82.25	5.37
24	N8.03	0.16	4.03	0.46	4.33	4.20	4.22	N5.15	3.61	79.51	8.63	84.52	81.53	4.76
25	N6.22	0.15	4.02	0.45	4.32	4.17	3.93	N3.16	2.56	78.46	8.39	84.28	79.89	3.38
26	N1.10	0.14	4.00	0.44	4.30	4.14	3.50	N3.19	2.45	78.34	8.16	84.05	79.72	3.23
27	N8.04	0.13	3.99	0.43	4.29	4.12	3.30	N2.13	2.38	78.28	7.94	83.84	79.61	3.14
28	N3.16	0.12	3.99	0.42	4.28	4.12	3.19	N3.15	2.38	78.27	7.74	83.64	79.60	3.13
29	N4.05	0.12	3.99	0.41	4.27	4.11	3.13	N1.19	2.28	78.18	7.56	83.45	79.45	3.01
30	N4.06	0.10	3.97	0.40	4.26	4.07	2.64	N4.07	2.27	78.16	7.38	83.27	79.43	2.99
31	N5.19	0.09	3.95	0.39	4.25	4.05	2.30	N3.08	2.24	78.13	7.21	83.11	79.38	2.95
32	N5.02	0.08	3.95	0.38	4.24	4.03	2.14	N5.21	1.82	77.71	7.05	82.94	78.73	2.40
33	N4.13	0.07	3.93	0.37	4.23	4.00	1.73	N5.18	1.75	77.64	6.88	82.78	78.62	2.30
34	N2.13	0.06	3.93	0.36	4.22	3.99	1.64	N1.22	1.62	77.51	6.73	82.62	78.42	2.13
35	N5.12	0.06	3.92	0.35	4.22	3.98	1.51	N2.16	1.50	77.39	6.58	82.47	78.23	1.97
36	N1.22	0.05	3.91	0.34	4.21	3.97	1.28	N4.21	1.40	77.30	6.44	82.33	78.08	1.85
37	N3.19	0.05	3.91	0.33	4.20	3.96	1.27	N5.03	1.19	77.08	6.29	82.19	77.74	1.56
38	N1.06	0.01	3.88	0.33	4.19	3.89	0.27	N2.01	0.86	76.75	6.15	82.05	77.23	1.13
39	N3.02	0.01	3.87	0.32	4.18	3.88	0.19	N5.14	0.83	76.72	6.02	81.91	77.19	1.09
40	N1.19	0.01	3.87	0.31	4.17	3.88	0.18	N3.02	0.59	76.49	5.88	81.77	76.82	0.78
41	N5.21	0.00	3.86	0.30	4.17	3.86	−0.04	N1.09	0.28	76.17	5.74	81.64	76.33	0.37
42	N5.03	−0.01	3.86	0.29	4.16	3.85	−0.19	N4.15	−0.53	75.37	5.59	81.49	75.07	−0.70
43	N3.12	−0.01	3.85	0.29	4.15	3.84	−0.27	N5.20	−0.63	75.27	5.45	81.34	74.92	−0.82
44	N3.05	−0.01	3.85	0.28	4.15	3.84	−0.33	N2.10	−0.94	74.95	5.30	81.20	74.42	−1.24
45	N3.08	−0.02	3.84	0.27	4.14	3.82	−0.59	N6.12	−0.99	74.90	5.16	81.06	74.34	−1.31
46	N2.10	−0.04	3.83	0.27	4.13	3.79	−1.00	N8.03	−1.05	74.85	5.03	80.92	74.26	−1.38
47	N4.14	−0.06	3.81	0.26	4.13	3.75	−1.50	N1.10	−1.36	74.54	4.89	80.79	73.77	−1.79
48	N3.15	−0.07	3.80	0.25	4.12	3.72	−1.79	N4.20	−1.40	74.50	4.76	80.66	73.71	−1.84
49	N5.06	−0.09	3.78	0.25	4.11	3.69	−2.22	N1.17	−1.45	74.45	4.64	80.53	73.64	−1.91
50	N6.12	−0.10	3.76	0.24	4.10	3.66	−2.60	N5.09	−1.55	74.35	4.51	80.41	73.48	−2.04
51	N6.05	−0.14	3.72	0.23	4.10	3.57	−3.72	N2.22	−1.66	74.24	4.39	80.28	73.31	−2.18
52	N1.07	−0.15	3.71	0.22	4.09	3.56	−3.88	N5.19	−2.10	73.80	4.27	80.16	72.62	−2.76
53	N1.17	−0.15	3.71	0.22	4.08	3.55	−3.96	N6.08	−2.11	73.79	4.15	80.04	72.61	−2.78
54	N2.22	−0.16	3.71	0.21	4.08	3.54	−4.06	N6.19	−2.39	73.51	4.02	79.92	72.17	−3.14
55	N3.22	−0.21	3.66	0.20	4.07	3.44	−5.35	N4.13	−2.82	73.07	3.90	79.79	71.49	−3.72
56	N4.15	−0.21	3.66	0.20	4.06	3.44	−5.36	N5.05	−2.94	72.95	3.78	79.67	71.30	−3.88
57	N5.22	−0.22	3.64	0.19	4.05	3.41	−5.73	N4.05	−3.09	72.80	3.66	79.55	71.07	−4.07
58	N4.20	−0.23	3.64	0.18	4.05	3.40	−5.88	N5.22	−3.70	72.19	3.53	79.42	70.12	−4.87
59	N5.16	−0.23	3.63	0.17	4.04	3.40	−5.96	N6.18	−4.00	71.89	3.40	79.30	69.65	−5.27
60	N3.07	−0.24	3.63	0.17	4.03	3.38	−6.17	N5.16	−4.01	71.89	3.28	79.17	69.64	−5.28
61	N2.01	−0.24	3.62	0.16	4.03	3.37	−6.28	N5.10	−4.34	71.55	3.15	79.05	69.12	−5.72
62	N6.18	−0.25	3.61	0.15	4.02	3.36	−6.47	N2.17	−4.43	71.46	3.03	78.93	68.98	−5.84
63	N5.14	−0.25	3.61	0.15	4.01	3.35	−6.56	N6.05	−4.70	71.19	2.91	78.80	68.56	−6.20
64	N2.17	−0.28	3.59	0.14	4.01	3.30	−7.21	N1.14	−4.72	71.17	2.79	78.68	68.53	−6.22
65	N4.21	−0.28	3.59	0.13	4.00	3.30	−7.22	N7.20	−4.76	71.13	2.67	78.57	68.46	−6.27
66	N1.13	−0.32	3.55	0.13	3.99	3.22	−8.24	N5.02	−5.26	70.64	2.55	78.45	67.69	−6.92
67	N1.14	−0.32	3.54	0.12	3.99	3.21	−8.27	N3.22	−5.48	70.41	2.43	78.33	67.34	−7.22
68	N1.12	−0.34	3.52	0.11	3.98	3.17	−8.81	N1.16	−5.68	70.22	2.31	78.21	67.03	−7.48
69	N1.16	−0.34	3.52	0.11	3.97	3.16	−8.92	N1.12	−5.75	70.14	2.20	78.09	66.92	−7.58
70	N7.20	−0.35	3.52	0.10	3.97	3.16	−8.98	N6.01	−5.77	70.13	2.08	77.98	66.90	−7.60
71	N2.18	−0.37	3.50	0.09	3.96	3.12	−9.47	N3.07	−6.09	69.80	1.97	77.86	66.39	−8.03
72	N1.09	−0.37	3.49	0.09	3.95	3.10	−9.67	N4.10	−6.24	69.65	1.85	77.75	66.16	−8.22
73	N6.19	−0.40	3.46	0.08	3.95	3.04	−10.47	N4.14	−6.59	69.30	1.74	77.63	65.61	−8.68
74	N4.12	−0.41	3.46	0.07	3.94	3.04	−10.53	N4.19	−6.98	68.92	1.62	77.51	65.01	−9.19
75	N5.10	−0.46	3.41	0.07	3.93	2.93	−11.81	N1.07	−7.16	68.73	1.50	77.40	64.72	−9.44
76	N6.01	−0.46	3.41	0.06	3.93	2.93	−11.83	N5.06	−7.78	68.11	1.38	77.28	63.76	−10.25
77	N5.11	−0.47	3.40	0.05	3.92	2.91	−12.14	N5.11	−9.61	66.28	1.24	77.13	60.90	−12.66
78	N7.15	−0.47	3.40	0.05	3.91	2.91	−12.14	N3.06	−9.69	66.20	1.10	76.99	60.78	−12.77
79	N4.19	−0.48	3.38	0.04	3.90	2.88	−12.54	N1.13	−10.11	65.78	0.96	76.85	60.12	−13.33
80	N4.10	−0.53	3.34	0.03	3.90	2.79	−13.64	N7.15	−12.82	63.07	0.79	76.68	55.89	−16.90
81	N5.05	−0.53	3.33	0.03	3.89	2.78	−13.77	N1.15	−13.59	62.31	0.61	76.50	54.70	−17.90
82	N4.17	−0.64	3.23	0.02	3.88	2.57	−16.49	N2.18	−14.41	61.49	0.42	76.32	53.42	−18.98
83	N1.15	−0.66	3.20	0.01	3.87	2.51	−17.17	N4.12	−15.22	60.68	0.24	76.13	52.15	−20.05
84	N3.06	−0.83	3.04	0.00	3.86	2.18	−21.40	N4.17	−19.59	56.30	0.00	75.89	45.32	−25.82
					**LSR**					**CT**				
**Order**	**Ecotypes**	**g**	**u + g**	**Gain**	**µ_n_**	**u + g + gem**	**SG**	**Ecotypes**	**g**	**u + g**	**Gain**	**µ_n_**	**u + g + gem**	**SG**
1	N4.14	0.87	3.81	0.87	3.81	4.48	29.82	N1.09	0.82	3.85	0.82	3.85	4.39	27.14
2	N4.10	0.70	3.63	0.79	3.72	4.17	23.82	N1.13	0.59	3.62	0.71	3.73	4.01	19.43
3	N5.12	0.62	3.55	0.73	3.66	4.03	21.15	N1.12	0.42	3.45	0.61	3.64	3.73	13.93
4	N1.13	0.59	3.52	0.69	3.63	3.97	19.95	N1.07	0.39	3.42	0.56	3.58	3.67	12.83
5	N1.10	0.53	3.46	0.66	3.59	3.87	18.00	N8.03	0.29	3.32	0.50	3.53	3.51	9.53
6	N7.20	0.47	3.41	0.63	3.56	3.77	16.16	N1.14	0.26	3.28	0.46	3.49	3.45	8.43
7	N1.09	0.46	3.40	0.61	3.54	3.76	15.81	N1.16	0.26	3.28	0.43	3.46	3.45	8.43
8	N2.01	0.41	3.34	0.58	3.52	3.66	13.98	N7.15	0.26	3.28	0.41	3.44	3.45	8.43
9	N1.21	0.39	3.33	0.56	3.49	3.63	13.41	N3.22	0.22	3.25	0.39	3.42	3.40	7.32
10	N2.06	0.38	3.31	0.54	3.48	3.61	12.96	N4.21	0.22	3.25	0.37	3.40	3.40	7.32
11	N1.19	0.37	3.30	0.53	3.46	3.58	12.52	N2.01	0.19	3.22	0.36	3.38	3.34	6.22
12	N3.08	0.29	3.23	0.51	3.44	3.45	10.02	N1.15	0.19	3.22	0.34	3.37	3.34	6.22
13	N6.08	0.25	3.18	0.49	3.42	3.37	8.41	N1.06	0.19	3.22	0.33	3.36	3.34	6.22
14	N1.14	0.24	3.18	0.47	3.40	3.36	8.26	N8.04	0.16	3.18	0.32	3.35	3.29	5.13
15	N5.22	0.23	3.16	0.45	3.39	3.34	7.84	N3.19	0.16	3.18	0.31	3.34	3.29	5.13
16	N2.18	0.22	3.15	0.44	3.37	3.32	7.51	N5.05	0.16	3.18	0.30	3.33	3.29	5.13
17	N4.12	0.21	3.15	0.43	3.36	3.31	7.25	N6.19	0.16	3.18	0.29	3.32	3.29	5.13
18	N1.15	0.21	3.14	0.41	3.35	3.31	7.15	N1.10	0.16	3.18	0.28	3.31	3.29	5.13
19	N2.07	0.20	3.14	0.40	3.34	3.29	6.92	N1.19	0.12	3.15	0.27	3.30	3.23	4.02
20	N3.02	0.19	3.13	0.39	3.33	3.27	6.56	N4.06	0.12	3.15	0.27	3.29	3.23	4.02
21	N5.14	0.19	3.12	0.38	3.32	3.27	6.46	N1.17	0.09	3.12	0.26	3.29	3.18	2.92
22	N5.06	0.19	3.12	0.37	3.31	3.26	6.32	N5.11	0.09	3.12	0.25	3.28	3.18	2.92
23	N4.21	0.16	3.09	0.36	3.30	3.21	5.34	N3.18	0.09	3.12	0.24	3.27	3.18	2.92
24	N1.12	0.15	3.08	0.36	3.29	3.20	5.08	N3.12	0.06	3.08	0.23	3.26	3.12	1.82
25	N6.22	0.13	3.07	0.35	3.28	3.17	4.58	N3.07	0.06	3.08	0.23	3.26	3.12	1.82
26	N3.04	0.13	3.06	0.34	3.27	3.16	4.42	N2.17	0.06	3.08	0.22	3.25	3.12	1.82
27	N3.19	0.12	3.06	0.33	3.26	3.15	4.16	N6.05	0.02	3.05	0.21	3.24	3.07	0.72
28	N6.19	0.12	3.05	0.32	3.26	3.14	4.07	N3.03	0.02	3.05	0.21	3.24	3.07	0.72
29	N7.15	0.11	3.04	0.32	3.25	3.12	3.63	N2.18	0.02	3.05	0.20	3.23	3.07	0.72
30	N5.19	0.08	3.01	0.31	3.24	3.07	2.60	N5.16	0.02	3.05	0.19	3.22	3.07	0.72
31	N4.13	0.08	3.01	0.30	3.23	3.07	2.59	N3.04	−0.01	3.02	0.19	3.22	3.01	−0.38
32	N3.16	0.08	3.01	0.29	3.23	3.07	2.57	N5.14	−0.01	3.02	0.18	3.21	3.01	−0.38
33	N5.10	0.07	3.01	0.29	3.22	3.06	2.50	N5.01	−0.01	3.02	0.18	3.20	3.01	−0.38
34	N4.08	0.07	3.00	0.28	3.21	3.06	2.35	N4.20	−0.01	3.02	0.17	3.20	3.01	−0.38
35	N5.21	0.06	3.00	0.27	3.21	3.05	2.19	N4.14	−0.01	3.02	0.16	3.19	3.01	−0.38
36	N3.22	0.06	3.00	0.27	3.20	3.05	2.17	N3.08	−0.01	3.02	0.16	3.19	3.01	−0.38
37	N3.15	0.05	2.99	0.26	3.20	3.03	1.80	N1.18	−0.01	3.02	0.16	3.18	3.01	−0.38
38	N8.04	0.04	2.97	0.26	3.19	3.00	1.34	N3.09	−0.01	3.02	0.15	3.18	3.01	−0.38
39	N5.02	0.03	2.97	0.25	3.18	2.99	1.11	N3.15	−0.01	3.02	0.15	3.18	3.01	−0.38
40	N6.12	0.03	2.96	0.24	3.18	2.98	0.92	N4.05	−0.01	3.02	0.14	3.17	3.01	−0.38
41	N5.20	0.03	2.96	0.24	3.17	2.98	0.86	N4.13	−0.04	2.98	0.14	3.17	2.95	−1.48
42	N3.18	0.02	2.95	0.23	3.17	2.96	0.53	N4.15	−0.04	2.98	0.13	3.16	2.95	−1.48
43	N6.01	−0.03	2.90	0.23	3.16	2.88	−0.99	N3.05	−0.04	2.98	0.13	3.16	2.95	−1.48
44	N5.09	−0.03	2.90	0.22	3.16	2.88	−1.08	N4.19	−0.04	2.98	0.13	3.15	2.95	−1.48
45	N3.06	−0.04	2.89	0.22	3.15	2.87	−1.30	N2.10	−0.04	2.98	0.12	3.15	2.95	−1.48
46	N3.17	−0.04	2.89	0.21	3.14	2.86	−1.34	N2.16	−0.04	2.98	0.12	3.15	2.95	−1.48
47	N1.22	−0.04	2.89	0.21	3.14	2.86	−1.43	N6.01	−0.04	2.98	0.11	3.14	2.95	−1.48
48	N4.05	−0.05	2.88	0.20	3.13	2.85	−1.65	N4.12	−0.04	2.98	0.11	3.14	2.95	−1.48
49	N2.11	−0.05	2.88	0.19	3.13	2.84	−1.77	N5.15	−0.04	2.98	0.11	3.14	2.95	−1.48
50	N4.07	−0.06	2.88	0.19	3.12	2.83	−1.98	N5.18	−0.04	2.98	0.11	3.13	2.95	−1.48
51	N1.06	−0.08	2.85	0.18	3.12	2.79	−2.68	N2.13	−0.04	2.98	0.10	3.13	2.95	−1.48
52	N6.05	−0.08	2.85	0.18	3.11	2.79	−2.84	N3.06	−0.04	2.98	0.10	3.13	2.95	−1.48
53	N5.18	−0.14	2.80	0.17	3.11	2.69	−4.62	N6.22	−0.04	2.98	0.10	3.13	2.95	−1.48
54	N5.03	−0.14	2.79	0.17	3.10	2.68	−4.87	N5.06	−0.08	2.95	0.09	3.12	2.90	−2.58
55	N3.07	−0.14	2.79	0.16	3.10	2.68	−4.89	N3.17	−0.08	2.95	0.09	3.12	2.90	−2.58
56	N4.20	−0.17	2.77	0.16	3.09	2.64	−5.67	N6.18	−0.08	2.95	0.09	3.12	2.90	−2.58
57	N5.11	−0.19	2.75	0.15	3.08	2.60	−6.37	N5.09	−0.08	2.95	0.08	3.11	2.90	−2.58
58	N2.17	−0.19	2.75	0.14	3.08	2.60	−6.38	N6.12	−0.08	2.95	0.08	3.11	2.90	−2.58
59	N3.10	−0.19	2.74	0.14	3.07	2.60	−6.44	N1.22	−0.08	2.95	0.08	3.11	2.90	−2.58
60	N3.03	−0.19	2.74	0.13	3.07	2.60	−6.49	N5.22	−0.08	2.95	0.08	3.11	2.90	−2.58
61	N2.02	−0.19	2.74	0.13	3.06	2.59	−6.59	N4.09	−0.11	2.92	0.07	3.10	2.84	−3.68
62	N3.12	−0.20	2.73	0.12	3.06	2.58	−6.76	N3.16	−0.11	2.92	0.07	3.10	2.84	−3.68
63	N4.06	−0.21	2.72	0.12	3.05	2.56	−7.25	N5.02	−0.14	2.88	0.07	3.10	2.79	−4.78
64	N1.17	−0.23	2.70	0.11	3.04	2.53	−7.81	N5.19	−0.14	2.88	0.06	3.09	2.79	−4.78
65	N4.09	−0.24	2.70	0.11	3.04	2.51	−8.10	N5.03	−0.14	2.88	0.06	3.09	2.79	−4.78
66	N5.05	−0.24	2.69	0.10	3.03	2.51	−8.16	N2.11	−0.14	2.88	0.06	3.09	2.79	−4.78
67	N5.16	−0.25	2.69	0.10	3.03	2.50	−8.40	N5.20	−0.14	2.88	0.05	3.08	2.79	−4.78
68	N1.05	−0.25	2.68	0.09	3.02	2.49	−8.51	N5.21	−0.14	2.88	0.05	3.08	2.79	−4.78
69	N1.07	−0.25	2.68	0.09	3.02	2.48	−8.68	N4.17	−0.14	2.88	0.05	3.08	2.79	−4.78
70	N6.18	−0.26	2.67	0.08	3.01	2.47	−8.82	N3.02	−0.14	2.88	0.05	3.07	2.79	−4.78
71	N4.15	−0.27	2.66	0.08	3.01	2.45	−9.36	N5.10	−0.18	2.85	0.04	3.07	2.73	−5.88
72	N4.17	−0.28	2.66	0.07	3.00	2.44	−9.41	N7.20	−0.18	2.85	0.04	3.07	2.73	−5.88
73	N4.19	−0.29	2.65	0.07	3.00	2.43	−9.71	N5.12	−0.18	2.85	0.04	3.07	2.73	−5.88
74	N3.05	−0.29	2.65	0.06	2.99	2.43	−9.73	N1.21	−0.18	2.85	0.03	3.06	2.73	−5.88
75	N8.03	−0.32	2.61	0.06	2.99	2.36	−11.04	N6.08	−0.21	2.82	0.03	3.06	2.68	−6.99
76	N2.16	−0.34	2.59	0.05	2.98	2.33	−11.59	N4.08	−0.21	2.82	0.03	3.06	2.68	−6.99
77	N5.01	−0.39	2.54	0.05	2.98	2.23	−13.43	N1.05	−0.21	2.82	0.02	3.05	2.68	−6.99
78	N1.16	−0.40	2.53	0.04	2.97	2.22	−13.73	N2.22	−0.21	2.82	0.02	3.05	2.68	−6.99
79	N1.18	−0.41	2.52	0.03	2.97	2.20	−14.14	N2.07	−0.21	2.82	0.02	3.05	2.68	−6.99
80	N3.09	−0.45	2.48	0.03	2.96	2.13	−15.39	N4.07	−0.24	2.78	0.01	3.04	2.62	−8.08
81	N5.15	−0.50	2.44	0.02	2.95	2.05	−16.92	N4.10	−0.24	2.78	0.01	3.04	2.62	−8.08
82	N2.22	−0.52	2.41	0.01	2.95	2.01	−17.68	N2.06	−0.28	2.75	0.01	3.04	2.57	−9.19
83	N2.10	−0.56	2.37	0.01	2.94	1.94	−19.18	N2.02	−0.31	2.72	0.00	3.03	2.51	−10.29
84	N2.13	−0.64	2.29	0.00	2.93	1.79	−21.89	N3.10	−0.34	2.68	0.00	3.03	2.46	−11.39
					**GH**					**PH (cm)**				
**Order**	**Ecotypes**	**g**	**u + g**	**Gain**	**µ_n_**	**u + g + gem**	**SG**	**Ecotypes**	**g**	**u + g**	**Gain**	**µ_n_**	**u + g + gem**	**SG**
1	N5.14	0.73	3.60	0.73	3.60	3.65	25.33	N2.02	2.97	59.99	2.97	59.99	65.46	5.21
2	N5.15	0.73	3.60	0.73	3.60	3.65	25.33	N1.18	2.09	59.11	2.53	59.55	62.96	3.66
3	N5.16	0.73	3.60	0.73	3.60	3.65	25.33	N3.09	1.71	58.73	2.26	59.28	61.89	3.00
4	N4.21	0.73	3.60	0.73	3.60	3.65	25.33	N2.06	1.71	58.73	2.12	59.14	61.88	3.00
5	N7.20	0.73	3.60	0.73	3.60	3.65	25.33	N2.10	1.32	58.34	1.96	58.98	60.77	2.31
6	N6.01	0.66	3.53	0.72	3.59	3.58	22.93	N5.09	1.18	58.20	1.83	58.85	60.38	2.07
7	N6.12	0.66	3.53	0.71	3.58	3.58	22.93	N1.21	1.16	58.18	1.73	58.76	60.32	2.03
8	N6.19	0.66	3.53	0.70	3.57	3.58	22.93	N3.02	1.12	58.14	1.66	58.68	60.20	1.96
9	N1.21	0.66	3.53	0.70	3.57	3.58	22.93	N4.07	1.03	58.05	1.59	58.61	59.94	1.80
10	N2.17	0.66	3.53	0.69	3.56	3.58	22.93	N3.10	0.90	57.92	1.52	58.54	59.58	1.58
11	N2.13	0.66	3.53	0.69	3.56	3.58	22.93	N5.15	0.86	57.88	1.46	58.48	59.47	1.51
12	N4.17	0.66	3.53	0.69	3.56	3.58	22.93	N5.22	0.80	57.82	1.40	58.43	59.29	1.40
13	N5.01	0.66	3.53	0.68	3.56	3.58	22.93	N2.16	0.78	57.80	1.36	58.38	59.24	1.37
14	N1.15	0.66	3.53	0.68	3.55	3.58	22.93	N4.08	0.75	57.77	1.31	58.33	59.15	1.31
15	N3.12	0.66	3.53	0.68	3.55	3.58	22.93	N8.04	0.75	57.77	1.28	58.30	59.15	1.31
16	N1.14	0.66	3.53	0.68	3.55	3.58	22.93	N2.07	0.74	57.76	1.24	58.26	59.13	1.30
17	N4.19	0.52	3.39	0.67	3.54	3.43	18.13	N5.01	0.72	57.74	1.21	58.23	59.06	1.26
18	N1.12	0.31	3.18	0.65	3.52	3.21	10.92	N1.06	0.64	57.66	1.18	58.20	58.83	1.12
19	N5.05	0.24	3.12	0.63	3.50	3.13	8.52	N1.10	0.60	57.62	1.15	58.17	58.72	1.05
20	N4.14	0.24	3.12	0.61	3.48	3.13	8.52	N3.08	0.56	57.59	1.12	58.14	58.62	0.99
21	N3.09	0.24	3.12	0.59	3.46	3.13	8.52	N1.13	0.55	57.57	1.09	58.11	58.59	0.97
22	N3.05	0.24	3.12	0.58	3.45	3.13	8.52	N6.22	0.55	57.57	1.07	58.09	58.59	0.97
23	N7.15	0.24	3.12	0.56	3.43	3.13	8.52	N2.01	0.55	57.57	1.04	58.07	58.58	0.96
24	N3.15	0.24	3.12	0.55	3.42	3.13	8.52	N3.12	0.55	57.57	1.02	58.05	58.58	0.96
25	N5.06	0.24	3.12	0.54	3.41	3.13	8.52	N3.17	0.55	57.57	1.01	58.03	58.57	0.96
26	N4.06	0.24	3.12	0.53	3.40	3.13	8.52	N1.05	0.44	57.46	0.98	58.01	58.27	0.77
27	N5.21	0.18	3.05	0.51	3.38	3.06	6.12	N3.04	0.40	57.42	0.96	57.98	58.15	0.70
28	N5.22	0.18	3.05	0.50	3.37	3.06	6.12	N3.07	0.38	57.40	0.94	57.96	58.10	0.67
29	N5.18	0.18	3.05	0.49	3.36	3.06	6.12	N5.06	0.33	57.35	0.92	57.94	57.96	0.58
30	N6.22	0.18	3.05	0.48	3.35	3.06	6.12	N4.13	0.31	57.34	0.90	57.92	57.91	0.55
31	N6.18	0.18	3.05	0.47	3.34	3.06	6.12	N4.10	0.30	57.32	0.88	57.90	57.86	0.52
32	N4.12	0.18	3.05	0.46	3.33	3.06	6.12	N1.07	0.25	57.27	0.86	57.88	57.72	0.43
33	N3.02	0.18	3.05	0.45	3.32	3.06	6.12	N2.22	0.22	57.24	0.84	57.86	57.65	0.39
34	N3.16	0.18	3.05	0.44	3.31	3.06	6.12	N5.05	0.22	57.24	0.82	57.84	57.64	0.38
35	N4.10	0.18	3.05	0.44	3.31	3.06	6.12	N2.17	0.22	57.24	0.81	57.83	57.64	0.38
36	N1.05	0.18	3.05	0.43	3.30	3.06	6.12	N4.12	0.21	57.24	0.79	57.81	57.63	0.37
37	N5.10	0.18	3.05	0.42	3.29	3.06	6.12	N5.19	0.21	57.24	0.77	57.80	57.63	0.37
38	N4.05	0.18	3.05	0.42	3.29	3.06	6.12	N4.05	0.18	57.20	0.76	57.78	57.54	0.32
39	N1.07	0.18	3.05	0.41	3.28	3.06	6.12	N4.09	0.09	57.11	0.74	57.76	57.28	0.16
40	N3.07	0.18	3.05	0.40	3.27	3.06	6.12	N4.20	0.09	57.11	0.72	57.75	57.27	0.15
41	N1.22	0.11	2.98	0.40	3.27	2.99	3.72	N4.17	0.06	57.08	0.71	57.73	57.18	0.10
42	N1.13	0.11	2.98	0.39	3.26	2.99	3.72	N7.20	0.02	57.04	0.69	57.71	57.08	0.04
43	N2.11	0.11	2.98	0.38	3.25	2.99	3.72	N3.06	0.02	57.04	0.68	57.70	57.07	0.03
44	N4.13	0.11	2.98	0.38	3.25	2.99	3.72	N3.03	0.01	57.03	0.66	57.68	57.04	0.01
45	N4.07	0.11	2.98	0.37	3.24	2.99	3.72	N4.14	−0.08	56.95	0.64	57.67	56.81	−0.13
46	N6.08	0.11	2.98	0.36	3.24	2.99	3.72	N4.06	−0.14	56.88	0.63	57.65	56.63	−0.24
47	N5.02	0.11	2.98	0.36	3.23	2.99	3.72	N1.22	−0.16	56.87	0.61	57.63	56.58	−0.27
48	N5.09	0.11	2.98	0.35	3.22	2.99	3.72	N5.12	−0.18	56.84	0.59	57.62	56.51	−0.32
49	N1.19	0.11	2.98	0.35	3.22	2.99	3.72	N6.08	−0.19	56.83	0.58	57.60	56.49	−0.33
50	N3.04	0.11	2.98	0.34	3.22	2.99	3.72	N5.20	−0.23	56.79	0.56	57.58	56.36	−0.41
51	N2.07	0.11	2.98	0.34	3.21	2.99	3.72	N1.19	−0.28	56.75	0.55	57.57	56.24	−0.48
52	N2.16	0.11	2.98	0.33	3.21	2.99	3.72	N5.02	−0.29	56.73	0.53	57.55	56.20	−0.51
53	N2.18	0.11	2.98	0.33	3.20	2.99	3.72	N1.12	−0.35	56.67	0.51	57.53	56.03	−0.61
54	N2.10	−0.17	2.70	0.32	3.19	2.69	−5.89	N5.14	−0.37	56.66	0.50	57.52	55.98	−0.64
55	N5.03	−0.24	2.63	0.31	3.18	2.62	−8.29	N3.18	−0.37	56.65	0.48	57.50	55.98	−0.65
56	N3.06	−0.24	2.63	0.30	3.17	2.62	−8.29	N5.16	−0.39	56.63	0.47	57.49	55.91	−0.69
57	N3.03	−0.31	2.56	0.29	3.16	2.54	−10.69	N3.05	−0.42	56.60	0.45	57.47	55.82	−0.74
58	N5.19	−0.31	2.56	0.28	3.15	2.54	−10.69	N4.19	−0.47	56.55	0.43	57.46	55.68	−0.83
59	N4.20	−0.31	2.56	0.27	3.14	2.54	−10.69	N1.09	−0.49	56.53	0.42	57.44	55.64	−0.86
60	N3.18	−0.31	2.56	0.26	3.13	2.54	−10.69	N5.18	−0.51	56.51	0.40	57.42	55.57	−0.90
61	N3.17	−0.31	2.56	0.25	3.12	2.54	−10.69	N2.11	−0.58	56.44	0.39	57.41	55.38	−1.02
62	N5.20	−0.31	2.56	0.24	3.11	2.54	−10.69	N5.03	−0.63	56.40	0.37	57.39	55.24	−1.10
63	N4.08	−0.38	2.50	0.23	3.10	2.47	−13.09	N3.22	−0.68	56.35	0.35	57.38	55.10	−1.19
64	N3.22	−0.38	2.50	0.22	3.09	2.47	−13.09	N3.16	−0.69	56.33	0.34	57.36	55.05	−1.22
65	N4.15	−0.38	2.50	0.21	3.08	2.47	−13.09	N3.15	−0.71	56.31	0.32	57.34	55.01	−1.24
66	N1.09	−0.38	2.50	0.20	3.08	2.47	−13.09	N3.19	−0.72	56.30	0.31	57.33	54.96	−1.27
67	N4.09	−0.44	2.43	0.20	3.07	2.39	−15.50	N1.17	−0.77	56.26	0.29	57.31	54.84	−1.34
68	N1.18	−0.44	2.43	0.19	3.06	2.39	−15.50	N8.03	−0.80	56.23	0.27	57.30	54.76	−1.39
69	N1.17	−0.44	2.43	0.18	3.05	2.39	−15.50	N2.18	−0.80	56.23	0.26	57.28	54.76	−1.39
70	N5.12	−0.44	2.43	0.17	3.04	2.39	−15.50	N7.15	−0.80	56.22	0.24	57.26	54.75	−1.40
71	N2.01	−0.44	2.43	0.16	3.03	2.39	−15.50	N6.19	−0.81	56.22	0.23	57.25	54.73	−1.41
72	N5.11	−0.44	2.43	0.15	3.02	2.39	−15.50	N6.05	−0.82	56.20	0.21	57.24	54.69	−1.44
73	N2.06	−0.72	2.15	0.14	3.01	2.10	−25.10	N5.21	−0.82	56.20	0.20	57.22	54.68	−1.44
74	N2.22	−0.72	2.15	0.13	3.00	2.10	−25.10	N1.15	−0.86	56.16	0.19	57.21	54.58	−1.51
75	N8.03	−0.79	2.08	0.12	2.99	2.03	−27.51	N4.15	−0.89	56.13	0.17	57.19	54.49	−1.56
76	N3.19	−0.86	2.01	0.10	2.97	1.95	−29.91	N2.13	−1.01	56.01	0.16	57.18	54.15	−1.77
77	N2.02	−0.93	1.94	0.09	2.96	1.88	−32.31	N6.12	−1.05	55.97	0.14	57.16	54.03	−1.85
78	N1.10	−0.93	1.94	0.08	2.95	1.88	−32.31	N5.10	−1.11	55.91	0.12	57.15	53.88	−1.94
79	N3.08	−0.93	1.94	0.06	2.93	1.88	−32.31	N5.11	−1.29	55.73	0.11	57.13	53.34	−2.27
80	N1.06	−1.00	1.87	0.05	2.92	1.80	−34.71	N1.16	−1.45	55.57	0.09	57.11	52.90	−2.54
81	N3.10	−1.00	1.87	0.04	2.91	1.80	−34.71	N6.01	−1.56	55.46	0.07	57.09	52.59	−2.74
82	N1.16	−1.00	1.87	0.02	2.90	1.80	−34.71	N6.18	−1.69	55.33	0.04	57.07	52.21	−2.97
83	N8.04	−1.00	1.87	0.01	2.88	1.80	−34.71	N4.21	−1.79	55.23	0.02	57.04	51.94	−3.14
84	N6.05	−1.00	1.87	0.00	2.87	1.80	−34.71	N1.14	−1.84	55.18	0.00	57.02	51.79	−3.23

g: genotypic effects; u + g: predicted genotypic values, free from all interaction with years; G: gain; µ_n_: new mean; u + g + gem: average genotypic value across the studied years, capturing the mean interaction with all evaluated environments; SG (%): individual selection gain relative to the overall mean of genotypes, expressed as a percentage. NT: number of tillers; FM: fresh matter; LDM: leaf dry matter; SDM: stem dry matter; IDM: inflorescence dry matter; TDM: total dry matter; LSR: leaf/stem ratio; CT: cold tolerance; GH: growth habit and PH: plant height.

## Appendix B

**Table A2:** Estimates of stability, adaptability, and genotypic performance for forage traits in 84 *P. nicorae* Parodi ecotypes.

**NT (Tillers Plant^−1^)**						**FM (g Plant^−1^)**					
**Ecotype**	**MHVG**	**PRVG**	**PRVG*µ**	**MHPRVG**	**MHPRVG*µ**	**Ecotype**	**MHVG**	**PRVG**	**PRVG*µ**	**MHPRVG**	**MHPRVG*µ**
N3.10	159.48	2.40	499.68	1.53	319.02	N3.10	67.13	1.79	278.14	1.42	220.60
N3.17	124.32	1.43	297.21	1.32	273.85	N2.06	77.58	1.38	214.81	1.31	203.89
N1.21	140.72	1.42	295.25	1.22	252.71	N4.09	60.76	1.30	201.99	1.27	197.44
N5.01	115.90	1.34	277.76	1.21	252.52	N1.21	78.37	1.44	223.82	1.27	196.44
N5.09	113.62	1.24	257.41	1.19	248.19	N1.05	72.52	1.32	205.49	1.24	192.90
N8.04	114.36	1.27	265.02	1.19	248.16	N5.01	66.08	1.32	204.50	1.24	192.64
N3.04	131.70	1.19	248.36	1.17	244.24	N3.09	55.73	1.25	194.02	1.17	180.88
N4.09	111.85	1.21	251.39	1.17	242.38	N1.06	65.84	1.31	203.36	1.13	175.70
N3.09	96.85	1.35	280.26	1.17	242.36	N4.08	59.24	1.17	182.31	1.13	175.31
N1.05	131.93	1.21	251.27	1.14	237.30	N3.16	58.03	1.13	174.69	1.11	172.48
N3.03	115.64	1.20	249.94	1.14	237.24	N2.07	61.42	1.17	181.21	1.09	168.45
N3.05	128.67	1.44	300.37	1.12	233.30	N2.02	57.95	1.13	174.97	1.08	168.00
N4.20	127.99	1.26	262.22	1.09	226.82	N3.05	59.99	1.22	189.83	1.08	167.43
N3.16	108.41	1.10	229.33	1.08	223.81	N1.22	52.48	1.09	169.65	1.07	166.78
N1.17	130.37	1.19	246.62	1.07	223.00	N2.16	52.36	1.12	173.25	1.06	164.70
N2.16	110.36	1.11	230.49	1.07	222.01	N3.04	53.08	1.17	181.30	1.05	162.56
N4.08	106.39	1.09	227.41	1.05	218.18	N3.12	50.26	1.10	170.37	1.05	162.34
N6.12	111.96	1.08	224.72	1.05	217.85	N2.11	55.34	1.14	176.53	1.03	160.49
N1.06	119.02	1.15	240.03	1.04	216.78	N2.10	55.25	1.05	163.42	1.03	159.94
N5.20	105.60	1.06	221.14	1.04	215.86	N3.02	62.88	1.14	176.34	1.03	159.45
N3.12	103.03	1.19	247.09	1.04	215.70	N1.19	73.63	1.36	211.24	1.02	158.79
N3.02	115.60	1.10	228.71	1.03	213.55	N3.19	49.33	1.04	161.96	1.02	157.72
N1.10	104.72	1.05	217.84	1.01	210.99	N8.04	51.22	1.13	176.03	1.02	157.59
N5.19	104.02	1.02	212.43	1.00	207.09	N4.06	51.01	1.06	164.78	1.00	155.86
N2.06	101.65	1.08	223.87	0.99	206.22	N1.17	52.87	1.07	165.40	1.00	155.80
N2.01	122.21	1.07	222.77	0.99	205.71	N3.03	45.89	1.03	159.30	1.00	155.70
N4.15	101.61	1.09	227.47	0.98	203.82	N3.17	54.60	1.17	181.73	1.00	155.57
N2.10	104.18	1.00	208.91	0.96	199.12	N1.18	53.69	1.05	163.56	1.00	154.73
N2.07	95.51	1.07	222.32	0.95	198.17	N5.12	47.56	1.16	179.76	0.98	152.77
N5.12	98.23	1.10	228.45	0.95	197.68	N6.08	56.56	1.03	159.44	0.97	150.48
N5.03	92.12	1.05	217.97	0.95	196.57	N2.01	52.69	1.01	155.96	0.97	150.11
N2.02	100.46	0.99	205.09	0.94	196.46	N5.19	57.53	1.03	159.92	0.96	149.21
N4.06	89.82	0.96	200.32	0.94	194.89	N5.22	55.98	0.99	152.94	0.96	148.97
N2.11	92.13	1.05	218.80	0.93	193.04	N4.21	54.56	1.01	157.36	0.96	148.49
N5.21	103.26	0.94	195.09	0.92	190.79	N5.15	48.76	1.02	157.75	0.95	148.14
N4.21	106.70	0.98	203.50	0.91	188.42	N6.19	51.94	0.97	150.97	0.95	148.02
N3.15	98.48	0.96	198.62	0.90	188.09	N6.22	63.59	1.09	168.80	0.94	146.36
N5.15	96.11	0.92	192.18	0.90	186.55	N6.12	44.46	0.99	153.51	0.93	145.04
N1.19	115.12	1.06	220.68	0.89	185.51	N2.13	47.32	0.98	151.29	0.92	143.32
N4.13	90.30	0.96	199.82	0.89	184.31	N3.15	48.86	1.01	156.49	0.92	142.44
N5.05	91.74	0.91	189.37	0.88	183.59	N5.03	45.22	1.00	155.64	0.91	141.92
N4.07	88.21	0.98	203.69	0.88	183.52	N5.20	44.42	0.97	150.56	0.90	140.20
N2.13	88.52	0.92	191.09	0.86	179.55	N5.21	39.96	0.91	140.98	0.89	138.22
N1.18	89.11	0.97	201.91	0.86	178.54	N3.18	47.37	1.06	164.22	0.89	137.96
N5.22	89.97	0.93	193.15	0.86	178.43	N4.15	48.50	0.93	144.77	0.88	137.21
N4.10	94.85	0.89	185.05	0.86	178.17	N4.13	41.20	0.95	147.19	0.88	136.77
N1.13	102.18	0.91	189.86	0.86	177.91	N6.18	42.82	0.89	138.45	0.87	134.33
N2.17	91.68	0.91	188.70	0.85	177.56	N4.20	40.85	0.92	143.33	0.86	133.93
N1.22	93.89	0.92	191.24	0.85	176.19	N5.18	40.09	0.99	153.39	0.84	130.52
N1.07	94.71	0.88	182.80	0.84	175.53	N5.09	36.81	0.96	149.45	0.83	128.73
N6.05	99.16	0.90	186.35	0.84	174.61	N6.05	56.18	1.19	184.89	0.82	127.49
N3.07	86.48	0.89	185.20	0.84	174.59	N3.08	47.21	0.93	144.98	0.82	126.98
N5.10	89.63	0.85	177.74	0.84	174.09	N5.14	35.78	0.86	134.00	0.82	126.56
N3.18	78.63	0.98	204.61	0.83	171.94	N1.16	47.53	0.87	134.27	0.81	126.42
N6.01	90.21	0.90	187.81	0.82	171.52	N5.06	40.23	0.83	128.32	0.81	125.86
N5.16	91.12	0.98	203.40	0.82	169.51	N5.02	42.23	0.85	131.93	0.81	125.48
N1.16	95.41	0.84	175.54	0.81	168.79	N5.10	37.52	0.88	136.66	0.81	125.14
N3.19	86.72	0.84	175.15	0.81	167.81	N4.07	43.00	0.97	150.02	0.80	124.48
N5.14	89.81	0.83	172.09	0.81	167.48	N7.20	40.43	0.84	129.66	0.79	122.86
N5.11	92.09	0.84	174.47	0.79	164.23	N1.10	38.05	0.88	135.91	0.78	121.48
N5.02	84.15	0.81	168.68	0.78	162.38	N4.05	39.99	0.90	139.48	0.78	121.41
N5.18	78.01	0.97	201.03	0.77	160.16	N4.10	39.40	0.83	129.28	0.78	120.99
N6.19	83.10	0.86	179.35	0.76	157.44	N5.16	42.27	0.94	146.32	0.78	120.86
N4.12	80.81	0.81	168.46	0.75	155.05	N5.05	39.54	0.85	132.43	0.77	120.01
N5.06	80.74	0.77	160.74	0.74	154.58	N4.19	42.79	0.80	123.63	0.77	119.58
N3.22	77.17	0.82	171.55	0.74	154.15	N8.03	31.25	0.83	128.37	0.76	118.63
N8.03	80.49	0.79	164.06	0.74	154.10	N1.14	53.62	0.90	139.01	0.76	117.32
N1.12	98.59	0.90	187.83	0.74	153.30	N6.01	45.77	0.88	137.10	0.76	117.27
N1.15	86.77	0.80	165.48	0.73	151.43	N2.17	40.61	0.83	128.26	0.74	114.90
N7.20	75.00	0.83	172.45	0.72	150.34	N3.07	36.46	0.85	132.57	0.73	113.83
N1.14	100.90	0.92	191.92	0.70	145.34	N1.07	47.25	0.84	130.02	0.72	112.00
N1.09	80.93	1.06	220.59	0.68	142.09	N4.14	38.77	0.82	127.78	0.72	111.66
N4.14	79.63	0.87	180.42	0.68	140.62	N3.22	39.17	0.84	130.08	0.71	109.96
N4.05	69.16	0.90	187.87	0.67	139.14	N1.13	44.02	0.78	121.80	0.70	108.73
N6.18	77.00	0.72	148.81	0.66	137.83	N5.11	39.10	0.75	116.80	0.70	108.37
N6.08	68.61	0.81	168.41	0.65	134.62	N2.18	34.23	0.79	123.09	0.67	104.62
N4.19	68.48	0.75	155.01	0.63	130.52	N1.12	33.54	0.88	135.98	0.66	103.04
N6.22	65.19	0.87	179.93	0.60	123.78	N2.22	37.06	0.83	129.18	0.63	98.08
N3.08	66.15	0.72	150.09	0.59	122.55	N7.15	38.86	0.72	111.97	0.62	96.52
N2.22	56.93	0.91	189.43	0.57	118.69	N1.15	39.47	0.70	107.97	0.60	93.73
N2.18	55.99	0.71	147.71	0.55	114.86	N4.12	25.46	0.63	97.73	0.54	83.18
N3.06	55.74	0.71	148.38	0.53	111.22	N1.09	38.26	0.91	140.97	0.52	80.50
N7.15	70.49	0.64	134.00	0.49	101.25	N4.17	32.88	0.59	90.95	0.48	74.45
N4.17	58.07	0.57	119.34	0.46	95.50	N3.06	24.74	0.69	107.30	0.47	72.35
**LDM (g plant^−1^)**						**SDM (g plant^−1^)**					
**Ecotype**	**MHVG**	**PRVG**	**PRVG*µ**	**MHPRVG**	**MHPRVG*µ**	**Ecotype**	**MHVG**	**PRVG**	**PRVG*µ**	**MHPRVG**	**MHPRVG*µ**
N1.05	34.42	1.36	84.56	1.28	79.46	N2.06	4.86	1.49	9.73	1.40	9.17
N2.06	31.72	1.33	82.91	1.27	79.03	N3.10	3.83	1.84	12.02	1.19	7.76
N3.10	20.88	1.75	108.95	1.26	78.36	N5.01	3.50	1.21	7.93	1.13	7.38
N5.01	26.80	1.20	74.89	1.16	72.55	N3.17	3.71	1.50	9.79	1.12	7.31
N4.09	22.86	1.15	71.66	1.12	69.93	N3.05	2.82	1.21	7.93	1.11	7.25
N5.15	27.61	1.14	70.99	1.12	69.61	N3.09	3.14	1.23	8.06	1.10	7.20
N2.11	27.73	1.20	74.74	1.11	69.13	N1.21	3.11	1.21	7.91	1.08	7.09
N1.21	30.59	1.20	74.73	1.10	68.47	N3.04	2.51	1.21	7.93	1.08	7.07
N3.12	25.22	1.15	71.81	1.09	68.23	N6.22	3.71	1.20	7.84	1.08	7.05
N4.08	24.85	1.09	68.18	1.07	66.51	N2.01	3.44	1.13	7.37	1.07	7.04
N3.09	22.55	1.16	72.27	1.07	66.41	N4.09	3.20	1.18	7.72	1.06	6.94
N3.03	24.72	1.13	70.19	1.06	66.19	N5.21	2.54	1.12	7.30	1.04	6.78
N3.04	22.78	1.09	68.17	1.06	66.11	N5.19	3.96	1.14	7.46	1.02	6.65
N2.07	23.74	1.09	67.92	1.06	65.99	N5.15	2.63	1.10	7.21	1.01	6.63
N2.02	22.73	1.11	69.15	1.04	65.04	N1.22	2.85	1.05	6.85	1.01	6.62
N1.06	23.74	1.21	75.31	1.04	64.71	N1.18	3.33	1.08	7.05	1.01	6.60
N2.16	21.83	1.06	66.16	1.03	64.33	N3.19	2.87	1.07	6.97	1.01	6.58
N1.22	23.34	1.03	64.39	1.02	63.87	N8.04	2.42	1.14	7.44	1.00	6.53
N3.17	23.86	1.19	73.97	1.01	63.23	N1.17	3.50	1.06	6.93	0.99	6.49
N1.18	23.03	1.06	65.77	1.01	63.19	N5.14	2.60	1.04	6.83	0.99	6.48
N6.22	28.01	1.11	68.91	1.01	62.89	N4.20	2.78	1.02	6.67	0.98	6.40
N3.02	27.90	1.08	67.41	1.00	62.32	N1.06	3.54	1.11	7.29	0.96	6.28
N5.14	22.94	1.08	67.41	0.99	61.75	N2.16	2.40	1.00	6.58	0.95	6.24
N8.04	20.15	1.18	73.49	0.98	61.29	N4.13	2.86	1.11	7.27	0.95	6.22
N3.19	21.58	1.01	63.12	0.98	61.24	N3.02	3.55	1.12	7.36	0.95	6.20
N3.08	26.90	1.11	69.11	0.98	61.17	N2.07	3.38	1.01	6.62	0.94	6.18
N4.15	26.96	1.03	64.27	0.98	61.11	N2.11	2.87	1.01	6.62	0.94	6.15
N8.03	21.31	1.00	62.11	0.97	60.48	N1.05	3.84	1.20	7.83	0.94	6.14
N5.21	18.48	1.01	63.14	0.97	60.35	N3.16	2.32	1.02	6.68	0.94	6.12
N4.06	22.86	1.02	63.65	0.96	59.67	N4.15	3.02	0.99	6.47	0.92	6.05
N3.16	24.14	0.97	60.62	0.96	59.52	N3.18	3.03	1.06	6.94	0.92	6.04
N2.10	22.99	0.97	60.43	0.95	59.49	N4.08	2.95	0.98	6.43	0.91	5.96
N3.15	22.26	1.01	63.20	0.95	59.40	N5.12	3.21	1.10	7.21	0.91	5.94
N6.08	24.84	0.99	61.76	0.95	59.02	N3.08	2.73	1.00	6.54	0.90	5.92
N3.05	19.79	1.05	65.68	0.94	58.71	N4.06	2.88	1.14	7.46	0.90	5.92
N2.01	23.96	0.97	60.74	0.94	58.45	N1.19	3.97	1.35	8.84	0.89	5.82
N5.22	24.85	0.95	59.02	0.93	58.19	N3.12	2.55	1.06	6.92	0.88	5.77
N4.21	22.07	0.99	61.86	0.93	58.01	N3.03	2.41	0.98	6.44	0.87	5.70
N6.12	24.42	0.97	60.48	0.92	57.46	N3.15	2.71	1.03	6.76	0.87	5.69
N4.13	20.41	0.98	60.83	0.92	57.18	N5.03	2.23	1.00	6.56	0.87	5.68
N1.17	21.45	0.96	59.66	0.92	57.10	N5.10	2.26	0.96	6.26	0.86	5.65
N3.18	18.52	1.12	69.78	0.91	56.58	N2.02	2.10	0.99	6.48	0.86	5.65
N2.13	22.04	0.95	59.06	0.91	56.57	N4.21	3.27	0.96	6.26	0.86	5.61
N5.12	20.18	0.98	61.20	0.91	56.44	N4.07	2.36	0.94	6.15	0.85	5.56
N4.19	25.48	0.95	58.93	0.89	55.71	N8.03	2.49	0.91	5.93	0.83	5.44
N5.05	22.10	0.97	60.32	0.88	55.12	N2.17	2.57	1.00	6.53	0.83	5.41
N4.05	19.47	0.93	58.26	0.88	54.96	N6.08	2.79	0.89	5.83	0.82	5.38
N5.19	24.74	0.91	56.83	0.88	54.72	N6.19	2.94	0.94	6.18	0.82	5.35
N5.02	19.76	0.92	57.09	0.88	54.61	N5.22	2.98	0.89	5.80	0.82	5.35
N1.07	27.69	0.98	61.00	0.87	54.38	N5.06	2.32	0.93	6.09	0.82	5.34
N1.19	26.80	1.12	69.99	0.87	54.29	N4.10	2.60	0.87	5.73	0.81	5.30
N1.14	21.85	0.99	61.69	0.87	54.18	N1.14	2.16	0.88	5.74	0.81	5.30
N4.20	18.49	0.94	58.80	0.87	54.13	N2.10	2.66	0.96	6.30	0.81	5.30
N5.03	19.42	0.92	57.14	0.87	54.12	N5.18	1.97	0.98	6.41	0.80	5.27
N1.16	23.24	0.92	57.41	0.87	54.11	N1.10	1.81	0.87	5.69	0.80	5.26
N6.05	25.96	1.08	67.51	0.87	54.10	N2.13	2.02	0.91	5.96	0.80	5.23
N5.20	16.63	0.95	59.23	0.86	53.85	N4.19	3.11	0.87	5.71	0.80	5.22
N2.17	18.95	0.91	56.47	0.86	53.61	N4.14	2.45	0.91	5.98	0.79	5.18
N5.18	18.30	0.92	57.63	0.86	53.48	N7.20	2.49	0.87	5.70	0.79	5.17
N4.07	18.15	0.98	60.91	0.85	53.05	N5.09	2.31	0.93	6.10	0.78	5.12
N7.20	18.87	0.93	57.73	0.84	52.60	N1.09	2.12	0.96	6.25	0.78	5.08
N6.18	18.77	0.90	56.27	0.84	52.41	N3.22	2.19	0.92	5.99	0.77	5.07
N5.16	21.73	0.93	58.03	0.83	51.68	N6.05	3.22	1.15	7.54	0.77	5.03
N6.19	23.95	0.88	54.86	0.83	51.54	N6.12	2.93	0.99	6.48	0.76	5.00
N4.10	18.59	0.90	56.26	0.82	51.38	N1.16	2.00	0.87	5.71	0.76	5.00
N5.11	25.21	0.89	55.65	0.82	50.84	N5.02	2.06	0.84	5.49	0.75	4.93
N5.06	19.41	0.82	50.90	0.80	49.91	N5.05	2.30	0.77	5.07	0.74	4.86
N1.12	21.62	0.96	59.63	0.80	49.72	N6.18	2.14	0.81	5.29	0.74	4.83
N1.10	19.07	0.84	52.39	0.80	49.64	N6.01	2.91	0.88	5.75	0.73	4.81
N3.07	17.96	0.87	54.49	0.79	49.50	N5.20	1.72	0.82	5.40	0.73	4.79
N5.10	17.24	0.90	56.37	0.79	49.45	N4.05	1.65	0.81	5.32	0.72	4.68
N1.09	21.46	0.95	59.47	0.78	48.89	N1.13	2.98	0.80	5.26	0.71	4.66
N5.09	17.37	0.87	54.49	0.76	47.64	N1.07	1.84	0.86	5.63	0.71	4.66
N6.01	22.36	0.84	52.26	0.76	47.56	N3.07	2.19	0.82	5.36	0.69	4.50
N4.14	18.89	0.83	51.95	0.76	47.45	N7.15	2.30	0.76	4.96	0.67	4.40
N3.22	20.28	0.83	51.57	0.76	47.27	N4.12	1.64	0.76	4.99	0.67	4.39
N1.13	23.67	0.80	49.74	0.72	45.07	N1.12	1.57	0.84	5.51	0.67	4.37
N1.15	23.65	0.81	50.72	0.71	44.46	N2.22	2.48	1.02	6.65	0.66	4.33
N3.06	17.29	0.82	51.37	0.71	44.45	N5.16	2.23	0.76	4.98	0.66	4.33
N4.12	17.50	0.73	45.73	0.69	43.27	N5.11	2.47	0.72	4.73	0.66	4.30
N7.15	20.50	0.74	46.31	0.68	42.61	N2.18	1.32	0.79	5.19	0.66	4.29
N2.22	18.22	0.89	55.52	0.68	42.15	N3.06	2.11	0.78	5.09	0.60	3.94
N2.18	18.47	0.72	45.12	0.67	42.05	N1.15	1.96	0.72	4.69	0.58	3.80
N4.17	20.24	0.72	44.80	0.61	38.31	N4.17	1.24	0.61	3.96	0.47	3.08
**IDM (g plant^−1^)**						**TDM (g plant^−1^)**					
**Ecotype**	**MHVG**	**PRVG**	**PRVG*µ**	**MHPRVG**	**MHPRVG*µ**	**Ecotype**	**MHVG**	**PRVG**	**PRVG*µ**	**MHPRVG**	**MHPRVG*µ**
N3.10	2.47	1.84	7.12	1.36	5.24	N3.10	41.40	1.93	146.27	1.43	108.77
N1.05	2.15	1.34	5.19	1.23	4.76	N2.06	43.26	1.36	103.47	1.29	97.80
N1.21	2.81	1.48	5.70	1.21	4.68	N1.05	42.32	1.32	100.19	1.26	95.67
N5.15	2.00	1.21	4.70	1.18	4.54	N1.21	42.23	1.34	101.43	1.24	93.82
N3.04	2.47	1.38	5.32	1.18	4.54	N4.09	33.09	1.23	93.24	1.21	91.48
N4.09	1.81	1.18	4.54	1.12	4.32	N5.01	35.03	1.21	91.51	1.16	88.39
N2.16	1.92	1.25	4.84	1.11	4.30	N3.09	31.34	1.23	93.14	1.12	85.32
N2.02	2.35	1.29	4.97	1.10	4.26	N3.04	31.14	1.16	87.67	1.12	84.73
N2.11	1.88	1.23	4.75	1.10	4.24	N2.07	32.33	1.14	86.29	1.10	83.44
N2.07	2.18	1.32	5.11	1.08	4.18	N2.11	33.03	1.16	87.71	1.09	82.91
N2.06	1.67	1.15	4.46	1.08	4.16	N1.06	35.59	1.25	94.78	1.09	82.68
N1.18	1.76	1.16	4.50	1.07	4.14	N2.02	32.43	1.13	86.09	1.09	82.49
N3.09	1.84	1.25	4.82	1.06	4.10	N5.15	31.44	1.10	83.76	1.08	82.25
N3.03	2.04	1.19	4.59	1.06	4.09	N4.08	31.20	1.08	82.20	1.06	80.11
N3.17	2.16	1.27	4.92	1.06	4.09	N3.12	29.35	1.10	83.28	1.06	80.07
N4.08	1.85	1.13	4.36	1.02	3.96	N4.06	30.42	1.11	84.57	1.05	79.94
N5.01	2.04	1.23	4.74	1.01	3.89	N1.18	31.31	1.09	82.91	1.05	79.65
N6.08	1.83	1.14	4.41	1.00	3.86	N6.22	37.58	1.16	88.01	1.05	79.46
N6.22	1.57	1.09	4.21	0.98	3.78	N3.03	28.91	1.08	81.72	1.04	79.08
N3.02	1.70	1.08	4.16	0.97	3.76	N8.04	29.72	1.17	88.80	1.03	78.44
N5.19	1.65	0.99	3.84	0.95	3.69	N3.05	26.10	1.15	87.01	1.03	78.14
N3.18	1.49	1.23	4.74	0.95	3.69	N3.17	32.56	1.21	92.15	1.03	78.09
N8.03	1.61	1.04	4.00	0.95	3.66	N2.16	26.14	1.06	80.11	1.03	77.87
N8.04	1.80	1.15	4.45	0.95	3.66	N3.16	30.47	1.04	78.72	1.03	77.87
N1.22	1.41	1.03	3.97	0.94	3.62	N1.22	28.25	1.02	77.41	1.01	76.64
N4.07	1.60	1.13	4.38	0.92	3.57	N3.19	27.86	1.04	79.16	1.01	76.58
N1.06	1.83	1.18	4.58	0.92	3.56	N5.21	25.13	1.01	76.79	1.00	76.13
N3.16	1.44	0.99	3.82	0.92	3.56	N2.01	30.02	1.03	78.32	1.00	75.64
N1.07	2.07	1.04	4.00	0.92	3.56	N5.12	27.14	1.08	81.97	0.98	74.28
N5.21	1.39	0.97	3.76	0.91	3.52	N3.02	33.84	1.07	81.07	0.98	74.18
N5.20	1.47	1.05	4.04	0.91	3.51	N2.10	29.01	0.99	75.36	0.98	74.14
N2.10	1.37	0.97	3.75	0.91	3.51	N2.13	27.32	1.01	76.78	0.97	73.65
N1.17	1.83	1.01	3.89	0.91	3.50	N3.18	27.77	1.14	86.24	0.97	73.45
N3.08	1.80	1.06	4.08	0.90	3.49	N4.21	29.68	0.99	75.38	0.96	72.86
N2.13	1.30	0.99	3.83	0.89	3.45	N5.22	30.67	0.96	72.77	0.94	71.41
N3.12	1.40	0.97	3.75	0.89	3.43	N5.14	25.06	1.00	76.14	0.93	70.71
N5.02	1.28	0.98	3.77	0.88	3.40	N4.15	29.73	0.98	74.18	0.93	70.65
N4.06	1.17	1.04	4.02	0.87	3.36	N3.08	30.18	1.04	79.27	0.93	70.64
N6.05	2.02	1.21	4.67	0.86	3.32	N3.15	27.04	1.00	75.81	0.93	70.60
N4.05	1.61	1.03	3.97	0.86	3.31	N1.17	26.47	0.98	74.03	0.92	70.13
N3.05	1.18	1.11	4.28	0.85	3.29	N6.08	29.00	0.96	72.87	0.92	69.64
N5.18	1.34	1.02	3.95	0.85	3.29	N8.03	22.88	0.94	70.97	0.91	69.22
N4.13	1.31	0.98	3.78	0.85	3.28	N5.19	30.12	0.95	72.24	0.91	68.95
N3.19	1.27	0.93	3.61	0.85	3.27	N5.03	23.54	0.97	73.46	0.91	68.92
N5.03	1.33	0.91	3.52	0.84	3.26	N5.18	22.12	0.99	75.32	0.90	68.02
N1.10	1.36	0.99	3.84	0.84	3.24	N4.13	22.83	0.95	72.24	0.88	67.11
N5.22	1.51	0.89	3.44	0.83	3.21	N6.12	26.98	0.93	70.83	0.88	66.67
N3.15	1.67	1.01	3.92	0.83	3.19	N4.20	23.57	0.93	70.23	0.88	66.42
N1.19	2.01	1.22	4.73	0.82	3.18	N5.02	24.03	0.91	69.02	0.87	66.27
N5.12	1.12	1.04	4.03	0.82	3.17	N6.19	28.66	0.92	69.67	0.87	65.84
N2.17	1.35	0.85	3.28	0.81	3.14	N5.20	20.81	0.94	71.39	0.86	65.25
N5.09	1.30	0.99	3.81	0.81	3.14	N4.07	24.28	0.99	75.09	0.86	65.20
N6.12	1.38	0.91	3.51	0.81	3.13	N1.14	27.56	0.97	73.76	0.86	65.14
N6.18	1.24	0.84	3.25	0.79	3.05	N1.19	34.74	1.16	87.83	0.86	64.92
N1.14	1.74	0.99	3.82	0.79	3.04	N2.17	23.29	0.91	68.81	0.85	64.71
N4.15	1.54	0.90	3.50	0.78	3.03	N6.18	23.55	0.88	66.69	0.85	64.50
N4.20	1.05	0.85	3.27	0.78	3.01	N1.10	22.77	0.91	68.80	0.85	64.45
N2.01	0.84	0.86	3.32	0.78	3.00	N4.05	21.37	0.91	69.17	0.84	63.69
N5.06	1.48	0.91	3.54	0.78	3.00	N4.19	26.58	0.87	65.98	0.84	63.67
N5.14	1.04	0.91	3.53	0.76	2.94	N7.20	23.07	0.89	67.49	0.84	63.51
N3.22	1.32	0.85	3.30	0.76	2.93	N5.05	23.07	0.89	67.73	0.83	63.12
N1.16	1.06	0.79	3.06	0.74	2.85	N4.10	22.34	0.86	65.45	0.82	62.01
N1.12	1.47	0.87	3.37	0.74	2.84	N6.05	29.78	1.05	80.00	0.82	61.91
N7.20	1.25	0.80	3.10	0.73	2.81	N1.16	25.21	0.87	65.81	0.81	61.49
N1.13	1.58	0.89	3.45	0.73	2.80	N1.07	28.76	0.90	67.99	0.81	61.44
N3.07	1.23	0.85	3.28	0.72	2.76	N5.06	22.00	0.81	61.37	0.79	60.27
N6.19	1.09	0.79	3.06	0.71	2.75	N5.09	19.99	0.91	69.26	0.79	59.98
N5.11	1.32	0.78	3.00	0.71	2.72	N6.01	26.05	0.87	66.19	0.79	59.82
N5.16	0.94	0.86	3.33	0.68	2.62	N3.07	21.11	0.84	63.91	0.78	59.07
N4.19	1.06	0.77	2.97	0.67	2.58	N5.16	24.23	0.89	67.29	0.78	58.98
N4.14	0.78	0.88	3.40	0.66	2.55	N3.22	22.58	0.86	65.08	0.77	58.60
N2.18	0.77	0.77	2.96	0.65	2.53	N5.10	20.55	0.87	66.08	0.77	58.45
N1.09	0.76	0.82	3.19	0.65	2.50	N5.11	25.52	0.80	60.50	0.75	56.73
N5.10	0.86	0.77	2.97	0.64	2.48	N4.14	21.33	0.82	62.40	0.74	55.95
N7.15	1.06	0.75	2.88	0.64	2.47	N1.09	25.43	0.94	71.23	0.73	55.55
N4.12	1.25	0.75	2.89	0.64	2.47	N1.13	27.03	0.81	61.42	0.73	55.41
N3.06	−1.38	0.54	2.07	0.61	2.35	N1.12	21.33	0.88	66.45	0.72	54.73
N2.22	0.83	0.78	3.03	0.60	2.33	N7.15	22.03	0.72	54.36	0.65	49.39
N4.10	0.72	0.76	2.94	0.60	2.30	N2.18	17.47	0.71	54.09	0.65	49.30
N1.15	0.96	0.62	2.40	0.57	2.22	N4.12	15.47	0.69	52.03	0.63	47.67
N5.05	0.63	0.69	2.66	0.57	2.19	N3.06	15.90	0.76	58.02	0.62	47.39
N4.21	0.38	0.86	3.32	0.56	2.18	N2.22	21.42	0.91	68.86	0.62	46.94
N4.17	0.79	0.67	2.59	0.52	1.99	N1.15	22.37	0.72	54.62	0.62	46.94
N6.01	0.09	0.72	2.76	0.25	0.97	N4.17	17.96	0.62	47.32	0.51	38.63
**LSR**						**CT**					
**Ecotype**	**MHVG**	**PRVG**	**PRVG*µ**	**MHPRVG**	**MHPRVG*µ**	**Ecotype**	**MHVG**	**PRVG**	**PRVG*µ**	**MHPRVG**	**MHPRVG*µ**
N1.13	3.13	1.35	3.96	1.28	3.74	N1.09	4.35	1.51	4.59	1.45	4.39
N5.12	2.92	1.33	3.91	1.23	3.61	N1.13	3.95	1.36	4.12	1.34	4.05
N1.21	2.83	1.23	3.61	1.15	3.38	N1.12	3.68	1.27	3.85	1.24	3.76
N4.10	2.61	1.34	3.94	1.15	3.37	N1.07	3.61	1.24	3.76	1.23	3.71
N2.01	2.67	1.21	3.56	1.14	3.34	N8.03	3.40	1.17	3.55	1.17	3.54
N7.20	2.63	1.23	3.62	1.10	3.23	N1.16	3.36	1.16	3.51	1.15	3.49
N2.07	2.77	1.15	3.36	1.08	3.17	N7.15	3.23	1.13	3.43	1.13	3.42
N4.21	2.65	1.10	3.22	1.07	3.14	N4.21	3.34	1.15	3.50	1.13	3.42
N5.14	2.56	1.10	3.23	1.06	3.12	N1.14	3.39	1.19	3.62	1.12	3.39
N3.02	2.54	1.11	3.24	1.05	3.09	N3.22	3.20	1.12	3.39	1.12	3.38
N5.22	2.55	1.13	3.31	1.05	3.07	N1.06	3.27	1.13	3.41	1.11	3.37
N4.14	2.42	1.42	4.15	1.04	3.04	N1.15	3.27	1.13	3.41	1.11	3.37
N2.06	2.52	1.20	3.53	1.04	3.04	N2.01	3.27	1.13	3.41	1.11	3.37
N5.06	2.56	1.10	3.24	1.04	3.04	N8.04	3.19	1.10	3.33	1.10	3.32
N6.19	2.48	1.06	3.11	1.03	3.02	N1.10	3.19	1.10	3.33	1.10	3.32
N1.14	2.45	1.11	3.26	1.03	3.01	N6.19	3.13	1.09	3.29	1.09	3.29
N1.09	2.53	1.24	3.63	1.02	3.00	N5.05	3.13	1.09	3.29	1.09	3.29
N2.18	2.56	1.11	3.27	1.02	2.99	N3.19	3.13	1.09	3.29	1.09	3.29
N3.19	2.52	1.07	3.13	1.01	2.98	N4.06	3.15	1.08	3.29	1.08	3.26
N1.12	2.40	1.06	3.11	1.01	2.95	N1.19	3.17	1.10	3.32	1.07	3.25
N8.04	2.51	1.03	3.04	1.01	2.95	N1.17	2.99	1.04	3.16	1.04	3.16
N3.16	2.43	1.04	3.05	1.00	2.94	N5.11	2.99	1.04	3.16	1.04	3.16
N1.10	2.42	1.26	3.69	1.00	2.94	N3.18	2.99	1.04	3.16	1.04	3.16
N3.04	2.55	1.10	3.22	1.00	2.93	N2.17	2.95	1.03	3.12	1.03	3.12
N5.10	2.38	1.02	3.00	1.00	2.93	N3.12	2.95	1.03	3.12	1.03	3.12
N3.18	2.52	1.02	3.00	1.00	2.92	N3.07	2.95	1.03	3.12	1.03	3.12
N3.15	2.36	1.01	2.96	0.99	2.92	N3.03	2.92	1.01	3.07	1.01	3.07
N1.15	2.44	1.11	3.26	0.99	2.91	N2.18	2.84	1.00	3.04	1.00	3.03
N6.08	2.38	1.11	3.25	0.99	2.89	N6.05	2.84	1.00	3.04	1.00	3.03
N3.08	2.38	1.14	3.33	0.99	2.89	N5.01	2.88	1.00	3.03	1.00	3.02
N5.21	2.36	1.03	3.01	0.97	2.86	N4.20	2.88	1.00	3.03	1.00	3.02
N3.22	2.38	1.03	3.02	0.97	2.84	N3.04	2.95	1.02	3.10	1.00	3.02
N5.09	2.40	0.99	2.89	0.97	2.84	N1.18	2.81	0.99	2.99	0.99	2.99
N1.19	2.37	1.18	3.46	0.97	2.83	N3.08	2.81	0.99	2.99	0.99	2.99
N5.19	2.39	1.04	3.06	0.96	2.82	N3.09	2.81	0.99	2.99	0.99	2.99
N5.20	2.37	1.01	2.97	0.96	2.82	N5.14	2.81	0.99	2.99	0.99	2.99
N2.11	2.40	0.98	2.87	0.96	2.81	N3.15	2.81	0.99	2.99	0.99	2.99
N7.15	2.41	1.07	3.15	0.96	2.81	N4.05	2.81	0.99	2.99	0.99	2.99
N4.13	2.36	1.04	3.04	0.96	2.80	N4.14	2.81	0.99	2.99	0.99	2.99
N3.06	2.37	0.98	2.88	0.95	2.79	N3.05	2.88	1.00	3.02	0.98	2.98
N5.02	2.35	1.02	2.99	0.95	2.78	N6.22	2.84	0.98	2.98	0.98	2.97
N4.12	2.39	1.11	3.25	0.94	2.76	N2.16	2.84	0.98	2.98	0.98	2.97
N1.22	2.39	0.99	2.90	0.94	2.75	N4.19	2.84	0.98	2.98	0.98	2.97
N1.06	2.31	0.96	2.81	0.93	2.73	N5.16	2.74	0.99	3.00	0.98	2.96
N4.05	2.33	0.99	2.90	0.92	2.70	N2.13	2.78	0.97	2.94	0.97	2.94
N6.12	2.28	1.01	2.95	0.92	2.69	N4.13	2.78	0.97	2.94	0.97	2.94
N4.08	2.29	1.03	3.02	0.91	2.68	N3.06	2.78	0.97	2.94	0.97	2.94
N6.01	2.25	0.97	2.86	0.90	2.65	N4.15	2.78	0.97	2.94	0.97	2.94
N5.18	2.22	0.92	2.70	0.90	2.63	N2.10	2.78	0.97	2.94	0.97	2.94
N5.03	2.28	0.94	2.75	0.89	2.61	N1.22	2.74	0.96	2.90	0.96	2.90
N3.07	2.19	0.91	2.67	0.89	2.61	N5.22	2.74	0.96	2.90	0.96	2.90
N4.07	2.24	0.97	2.85	0.89	2.61	N6.01	2.69	0.96	2.91	0.95	2.89
N6.05	2.25	0.96	2.80	0.89	2.60	N4.12	2.69	0.96	2.91	0.95	2.89
N3.12	2.25	0.91	2.67	0.88	2.58	N5.15	2.69	0.96	2.91	0.95	2.89
N6.22	2.21	1.07	3.13	0.88	2.57	N5.18	2.69	0.96	2.91	0.95	2.89
N3.10	2.15	0.89	2.61	0.87	2.56	N4.09	2.76	0.95	2.89	0.95	2.86
N3.03	2.23	0.91	2.66	0.87	2.54	N6.12	2.66	0.94	2.86	0.94	2.85
N2.17	2.20	0.90	2.65	0.86	2.53	N6.18	2.66	0.94	2.86	0.94	2.85
N4.06	2.24	0.90	2.65	0.86	2.52	N5.06	2.66	0.94	2.86	0.94	2.85
N1.07	2.21	0.88	2.59	0.85	2.51	N3.16	2.63	0.93	2.81	0.93	2.81
N5.11	2.18	0.91	2.66	0.85	2.48	N3.02	2.73	0.95	2.88	0.92	2.78
N4.20	2.09	0.90	2.64	0.83	2.43	N5.09	2.56	0.93	2.82	0.92	2.77
N1.17	2.12	0.88	2.59	0.83	2.43	N3.17	2.56	0.93	2.82	0.92	2.77
N3.05	2.20	0.87	2.57	0.83	2.42	N5.21	2.60	0.91	2.77	0.91	2.77
N6.18	2.12	0.87	2.54	0.82	2.40	N1.21	2.63	0.91	2.76	0.91	2.75
N2.02	2.07	0.89	2.61	0.82	2.40	N5.12	2.57	0.90	2.72	0.90	2.72
N4.15	2.04	0.84	2.47	0.81	2.37	N2.11	2.51	0.90	2.73	0.89	2.70
N1.05	2.08	0.87	2.55	0.81	2.36	N5.02	2.51	0.90	2.73	0.89	2.70
N4.19	2.04	0.85	2.48	0.80	2.33	N5.19	2.51	0.90	2.73	0.89	2.70
N2.16	2.01	0.81	2.38	0.79	2.32	N5.03	2.51	0.90	2.73	0.89	2.70
N4.17	2.12	0.88	2.59	0.79	2.31	N5.20	2.51	0.90	2.73	0.89	2.70
N8.03	2.00	0.82	2.40	0.79	2.30	N1.05	2.59	0.90	2.71	0.89	2.69
N3.17	1.97	0.96	2.82	0.77	2.26	N7.20	2.48	0.89	2.68	0.88	2.67
N1.16	2.05	0.81	2.37	0.76	2.23	N5.10	2.48	0.89	2.68	0.88	2.67
N4.09	1.95	0.86	2.51	0.76	2.23	N2.07	2.45	0.87	2.64	0.87	2.63
N5.16	1.94	0.87	2.55	0.74	2.18	N4.08	2.45	0.87	2.64	0.87	2.63
N3.09	1.91	0.76	2.22	0.74	2.16	N4.17	2.40	0.89	2.69	0.86	2.62
N5.01	1.89	0.78	2.29	0.74	2.16	N6.08	2.35	0.86	2.60	0.84	2.55
N5.05	1.92	0.88	2.59	0.71	2.07	N2.06	2.39	0.84	2.55	0.84	2.54
N1.18	1.85	0.78	2.28	0.70	2.06	N4.10	2.32	0.84	2.56	0.83	2.52
N5.15	1.81	0.73	2.14	0.69	2.04	N2.02	2.35	0.83	2.50	0.82	2.50
N2.22	1.77	0.72	2.12	0.67	1.97	N3.10	2.37	0.82	2.49	0.81	2.46
N2.10	1.68	0.71	2.09	0.61	1.79	N2.22	2.22	0.85	2.56	0.81	2.45
N2.13	1.59	0.67	1.96	0.57	1.68	N4.07	2.20	0.83	2.52	0.80	2.42
**GH**						**PH (cm)**					
**Ecotype**	**MHVG**	**PRVG**	**PRVG*µ**	**MHPRVG**	**MHPRVG*µ**	**Ecotype**	**MHVG**	**PRVG**	**PRVG*µ**	**MHPRVG**	**MHPRVG*µ**
N7.20	3.65	1.27	3.65	1.27	3.65	N2.02	64.21	1.15	65.46	1.15	65.37
N5.15	3.65	1.27	3.66	1.27	3.65	N1.18	61.11	1.10	62.65	1.10	62.56
N5.16	3.65	1.27	3.66	1.27	3.65	N3.09	61.53	1.09	62.39	1.09	62.11
N5.14	3.65	1.27	3.66	1.27	3.65	N2.06	60.73	1.09	61.87	1.08	61.81
N4.21	3.65	1.27	3.66	1.27	3.65	N2.10	60.12	1.07	61.02	1.07	60.93
N6.01	3.57	1.25	3.59	1.24	3.57	N5.09	59.55	1.06	60.54	1.06	60.44
N6.12	3.57	1.25	3.59	1.24	3.57	N3.02	58.99	1.06	60.16	1.05	60.11
N6.19	3.57	1.25	3.59	1.24	3.57	N1.21	58.83	1.05	60.13	1.05	60.09
N1.21	3.57	1.25	3.59	1.24	3.57	N4.07	59.13	1.05	60.08	1.05	60.05
N2.17	3.57	1.25	3.59	1.24	3.57	N5.15	58.83	1.05	59.71	1.05	59.64
N2.13	3.57	1.25	3.59	1.24	3.57	N3.10	58.48	1.05	59.59	1.04	59.50
N4.17	3.57	1.25	3.59	1.24	3.57	N2.16	58.36	1.04	59.41	1.04	59.20
N5.01	3.57	1.25	3.59	1.24	3.57	N2.07	58.23	1.04	59.25	1.04	59.13
N1.15	3.57	1.25	3.59	1.24	3.57	N5.22	57.95	1.04	59.17	1.04	59.11
N3.12	3.57	1.25	3.59	1.24	3.57	N4.08	57.91	1.04	59.13	1.03	58.93
N1.14	3.57	1.25	3.59	1.24	3.57	N8.04	57.69	1.03	58.99	1.03	58.93
N4.19	3.42	1.20	3.44	1.19	3.42	N1.06	58.01	1.03	59.00	1.03	58.85
N1.12	3.21	1.12	3.21	1.12	3.21	N5.01	57.87	1.04	59.08	1.03	58.84
N5.05	3.13	1.09	3.13	1.09	3.13	N3.12	57.82	1.03	58.73	1.03	58.71
N4.06	3.13	1.09	3.13	1.09	3.13	N1.13	57.84	1.03	58.75	1.03	58.70
N3.05	3.13	1.09	3.13	1.09	3.13	N3.08	57.72	1.03	58.71	1.03	58.66
N7.15	3.13	1.09	3.14	1.09	3.13	N3.17	57.68	1.03	58.65	1.03	58.64
N3.09	3.12	1.09	3.13	1.09	3.13	N2.01	57.65	1.03	58.63	1.03	58.62
N5.06	3.12	1.09	3.13	1.09	3.13	N5.06	57.49	1.02	58.31	1.02	58.17
N3.15	3.12	1.09	3.13	1.09	3.13	N3.07	57.21	1.02	58.18	1.02	58.17
N4.14	3.12	1.09	3.13	1.09	3.13	N3.04	57.15	1.02	58.19	1.02	58.15
N6.22	3.06	1.07	3.06	1.07	3.06	N1.05	56.72	1.02	58.05	1.02	57.96
N6.18	3.06	1.07	3.06	1.07	3.06	N4.10	57.00	1.02	57.95	1.02	57.93
N1.07	3.06	1.07	3.06	1.07	3.06	N1.07	57.25	1.02	58.07	1.02	57.92
N1.05	3.06	1.07	3.06	1.07	3.06	N2.22	57.21	1.02	58.00	1.02	57.87
N5.22	3.06	1.07	3.06	1.07	3.06	N4.13	56.65	1.01	57.80	1.01	57.79
N5.10	3.06	1.07	3.06	1.07	3.06	N5.05	56.82	1.01	57.75	1.01	57.73
N4.12	3.06	1.07	3.06	1.07	3.06	N5.19	56.66	1.01	57.65	1.01	57.65
N5.21	3.06	1.07	3.06	1.07	3.06	N4.12	56.48	1.01	57.58	1.01	57.53
N5.18	3.06	1.07	3.06	1.07	3.06	N1.10	55.80	1.02	58.18	1.01	57.46
N4.05	3.06	1.07	3.06	1.07	3.06	N2.17	56.32	1.01	57.51	1.01	57.45
N4.10	3.06	1.07	3.06	1.07	3.06	N4.05	56.13	1.01	57.38	1.01	57.33
N3.02	3.06	1.07	3.06	1.07	3.06	N4.17	56.46	1.01	57.34	1.01	57.31
N3.07	3.06	1.07	3.06	1.07	3.06	N3.06	56.14	1.00	57.12	1.00	57.11
N3.16	3.06	1.07	3.06	1.07	3.06	N4.20	56.12	1.00	57.27	1.00	57.10
N6.08	2.98	1.04	2.99	1.04	2.99	N3.03	56.13	1.00	57.11	1.00	57.06
N1.22	2.98	1.04	2.99	1.04	2.99	N4.09	56.02	1.00	57.23	1.00	57.04
N1.13	2.98	1.04	2.99	1.04	2.99	N4.06	56.39	1.00	57.15	1.00	56.87
N2.07	2.98	1.04	2.99	1.04	2.99	N4.14	56.07	1.00	57.02	1.00	56.86
N2.11	2.98	1.04	2.99	1.04	2.99	N7.20	55.66	1.00	56.90	1.00	56.86
N2.16	2.98	1.04	2.99	1.04	2.99	N6.22	54.58	1.01	57.69	0.99	56.62
N5.02	2.98	1.04	2.99	1.04	2.99	N1.22	55.50	0.99	56.55	0.99	56.54
N2.18	2.98	1.04	2.99	1.04	2.99	N5.12	55.14	0.99	56.35	0.99	56.32
N4.13	2.98	1.04	2.99	1.04	2.99	N1.19	55.22	0.99	56.23	0.99	56.23
N4.07	2.98	1.04	2.99	1.04	2.99	N5.20	55.07	0.99	56.26	0.99	56.18
N5.09	2.98	1.04	2.99	1.04	2.99	N1.12	54.98	0.98	56.01	0.98	56.00
N1.19	2.98	1.04	2.99	1.04	2.99	N5.02	54.59	0.98	55.94	0.98	55.84
N3.04	2.98	1.04	2.99	1.04	2.99	N4.19	55.12	0.98	55.96	0.98	55.82
N2.10	2.69	0.94	2.70	0.93	2.68	N6.08	54.33	0.98	56.03	0.98	55.79
N5.03	2.58	0.91	2.61	0.90	2.59	N3.05	54.29	0.98	55.63	0.97	55.49
N3.06	2.57	0.91	2.61	0.90	2.59	N5.14	54.09	0.98	55.68	0.97	55.42
N3.03	2.51	0.88	2.54	0.88	2.52	N5.18	54.29	0.97	55.45	0.97	55.41
N3.18	2.51	0.88	2.54	0.88	2.52	N1.09	54.92	0.98	56.13	0.97	55.37
N5.20	2.51	0.88	2.54	0.88	2.52	N3.22	54.91	0.98	55.66	0.97	55.32
N5.19	2.51	0.88	2.54	0.88	2.52	N3.16	54.58	0.97	55.40	0.97	55.20
N4.20	2.51	0.88	2.54	0.88	2.52	N3.19	54.43	0.97	55.22	0.97	55.14
N3.17	2.51	0.88	2.54	0.88	2.52	N1.17	54.19	0.97	55.04	0.96	54.97
N4.08	2.45	0.86	2.46	0.86	2.46	N5.16	53.36	0.97	55.37	0.96	54.95
N3.22	2.45	0.86	2.46	0.86	2.46	N5.03	54.36	0.98	55.60	0.96	54.94
N4.15	2.45	0.86	2.46	0.86	2.46	N8.03	54.13	0.96	54.95	0.96	54.91
N1.09	2.45	0.86	2.46	0.86	2.46	N7.15	54.13	0.96	54.98	0.96	54.86
N1.18	2.38	0.83	2.39	0.83	2.39	N3.18	53.14	0.97	55.31	0.96	54.80
N1.17	2.38	0.83	2.39	0.83	2.39	N6.05	53.93	0.96	54.80	0.96	54.79
N5.11	2.38	0.83	2.39	0.83	2.39	N6.19	53.92	0.96	54.84	0.96	54.77
N5.12	2.38	0.83	2.39	0.83	2.39	N1.15	53.88	0.96	54.74	0.96	54.70
N4.09	2.38	0.83	2.39	0.83	2.39	N4.15	53.85	0.96	54.80	0.96	54.49
N2.01	2.38	0.83	2.39	0.83	2.39	N3.15	53.07	0.96	54.64	0.96	54.45
N2.22	1.98	0.73	2.08	0.70	2.00	N2.11	52.77	0.96	54.79	0.95	54.38
N2.06	1.97	0.73	2.08	0.69	1.99	N5.21	52.53	0.95	54.31	0.95	53.92
N8.03	1.92	0.70	2.01	0.67	1.93	N2.13	52.91	0.95	54.14	0.95	53.90
N3.19	1.86	0.68	1.94	0.66	1.88	N5.10	53.09	0.95	54.01	0.95	53.89
N2.02	1.80	0.65	1.87	0.63	1.82	N2.18	52.19	0.95	54.18	0.94	53.76
N3.08	1.80	0.65	1.87	0.63	1.82	N5.11	52.61	0.94	53.47	0.94	53.42
N1.10	1.80	0.65	1.87	0.63	1.82	N6.12	51.25	0.94	53.39	0.93	52.89
N1.06	1.74	0.62	1.79	0.61	1.75	N1.16	51.89	0.93	52.87	0.93	52.87
N1.16	1.74	0.62	1.79	0.61	1.75	N6.01	51.83	0.92	52.70	0.92	52.63
N3.10	1.74	0.62	1.79	0.61	1.75	N4.21	51.17	0.91	52.03	0.91	51.99
N8.04	1.74	0.62	1.79	0.61	1.75	N1.14	51.23	0.91	52.00	0.91	51.93
N6.05	1.74	0.62	1.79	0.61	1.75	N6.18	50.40	0.91	51.85	0.91	51.69

NT: number of tillers; FM: fresh matter; LDM: leaf dry matter; SDM: stem dry matter; IDM: inflorescence dry matter; TDM: total dry matter; LSR: leaf/stem ratio; CT: cold tolerance; GH: growth habit and PH: plant height. MHVG—harmonic mean of genotypic values; PRVG—relative performance of genotypic values; PRVGμ—genotypic value capitalizing on adaptability; MHPRVG—harmonic mean of relative performance of genotypic values; MHPRVGμ—genotypic value penalized by instability and capitalized by adaptability.

## References

[1] Bengtsson J., Bullock J.M., Egoh B., Everson C., Everson T., O’Connor T., O’Farrell P.J., Smith H.G., Lindborg R. (2019). Grasslands—More Important for Ecosystem Services than You Might Think. Ecosphere.

[2] Matta F.D.P., Fávero A.P., Vigna B.B.Z., Cavallari M.M., Alves F., Oliveira F.A.D., Souza A.P.D., Pozzobon M.T., Azevedo A.L.S., Gusmão M.R. (2024). Characterization of *Paspalum* Genotypes for Turfgrass Cultivars Development. Crop Sci..

[3] Zuloaga F.O., Morrone O. (2005). Revisión de las Especies de Paspalum para América del Sur Austral (Argentina, Bolivia, Sur del Brasil, Chile, Paraguay y Uruguay).

[4] Valls J.F.M., Maciel J.R., Sousa M.W.D.S., Oliveira R.C., Pimenta K.M., Rua G.H. (2023). Paspalum. Flora e Funga do Brasil.

[5] Bonasora M.G., Pozzobon M.T., Honfi A.I., Rua G.H. (2015). *Paspalum schesslii* (Poaceae, Paspaleae), a New Species from Mato Grosso (Brazil) with an Unusual Base Chromosome Number. Plant Syst. Evol..

[6] Chase A. (1929). The North American Species of Paspalum.

[7] Judziewicz E.J., Görts-van Rijn A.R.A. (1990). Family 187. Poaceae (Gramineae). Flora of the Guianas. Series A: Phanerogams.

[8] Maciel J.R., Oliveira R.C., Alves M. (2009). *Paspalum* L. (Poaceae: Panicoideae: Paniceae) no Estado de Pernambuco, Brasil. Acta Bot. Bras..

[9] Denham S.S., Zuloaga F.O., Morrone O. (2002). Systematic Revision and Phylogeny of *Paspalum* subgenus *Ceresia* (Poaceae: Panicoideae: Paniceae). Ann. Mo. Bot. Gard..

[10] Rua G.H., Valls J.F.M., Graciano-Ribeiro D., Oliveira R.C. (2008). Four New Species of *Paspalum* (Poaceae, Paniceae) from Central Brazil, and Resurrection of an Old One. Syst. Bot..

[11] Pereira E.A., Dall’Agnol M., Nabinger C., Huber K.G.C., Montardo D.P., Genro T.C.M. (2011). Produção Agronômica de uma Coleção de Acessos de *Paspalum nicorae* Parodi. Rev. Bras. Zootec..

[12] Oliveira R.C., Valls J.F.M. (2015). Paspalum. Lista de Espécies da Flora do Brasil.

[13] Filho C.C.F., Barrios S.C.L., Santos M.F., Nunes J.A.R., do Valle C.B., Jank L., Rios E.F. (2025). Assessing Genotype Adaptability and Stability in Perennial Forage Breeding Trials Using Random Regression Models for Longitudinal Dry Matter Yield Data. G3 Genes Genomes Genet..

[14] Cook B.G., Lloyd D.L., Scattini W.J., Robertson A.D. (2024). *Paspalum lepton*—A Valuable Adjunct to the Suite of Grasses Used in Grazing Systems in the Subtropics or a Potential Weed. Trop. Grassl.-Forrajes Trop..

[15] Machado J.M., Dall’Agnol M., da Motta E.A.M., Pereira E.A., Simioni C., Weiler R.L., Zuñeda M.P., Ferreira P.B. (2017). Agronomic Evaluation of *Paspalum notatum* Flügge under the Influence of Photoperiod. Rev. Bras. Zootec..

[16] Motta E.A.M.d., Dall’Agnol M., Pereira E.A., Machado J.M., Simioni C. (2017). Valor Forrageiro de Híbridos Interespecíficos Superiores de *Paspalum*. Rev. Cienc. Agron..

[17] Weiler R.L., Dall’Agnol M., Simioni C., Krycki K.C., Pereira E.A., Machado J.M., Motta E.A.M.d. (2018). Intraspecific Tetraploid Hybrids of *Paspalum notatum*: Agronomic Evaluation of Segregating Progeny. Sci. Agric..

[18] Saraiva K.M., Dall’Agnol M., da Motta E.A., Pereira E.A., de Souza C.H., Simioni C., Weiler R.L., Kopp M.M., Schneider-Canny R., Barbosa M.R. (2021). Hybrids of *Paspalum plicatulum* × *P. guenoarum*: Selection for Forage Yield and Cold Tolerance in a Subtropical Environment. Trop. Grassl.-Forrajes Trop..

[19] Krycki K.C., da Motta E.A.M., Brunes A.P., Mills A., Weiler R.L., Simioni C. (2022). Hybrid Progenies of Bahiagrass: Agronomic Evaluation. Sci. Agrar. Parana..

[20] Eeuwijk F.A., Bustos-Korts D.V., Malosetti M. (2016). What Should Students in Plant Breeding Know about the Statistical Aspects of Genotype × Environment Interactions?. Crop Sci..

[21] Marcón F., Brugnoli E.A., Nunes J.A.R., Gutierrez V.A., Martínez E.J., Acuña C.A. (2021). Evaluating General Combining Ability for Agro-Morphological Traits in Tetraploid Bahiagrass. Euphytica.

[22] Silveira D.C., Machado J.M., da Motta E.A.M., Barbosa M.R., Simioni C., Weiler R.L., Mills A., Sampaio R., Brunes A.P., Dall’Agnol M. (2022). Genetic Parameters, Prediction of Gains and Intraspecific Hybrid Selection of *Paspalum notatum* Flügge for Forage Using REML/BLUP. Agronomy.

[23] Silveira D.C., Weiler R.L., Brunes A.P., Simioni C., Mills A., Longhi J., Corrêa M.V.S., Nauderer C., Valentini A., dos Santos W.M. (2023). REML/BLUP Methodology for Selection of Intraspecific Hybrids of *Paspalum notatum* Flügge by Multivariate Analysis. An. Acad. Bras. Cienc..

[24] Olivoto T., Lúcio A.D., Silva J.A.d., Marchioro V.S., Souza V.Q.d., Jost E. (2019). Mean Performance and Stability in Multi-Environment Trials I: Combining Features of AMMI and BLUP Techniques. Agron. J..

[25] Viana A.P., Resende M.D.V. (2014). Genética Quantitativa no Melhoramento de Fruteiras.

[26] Olivoto T., Lúcio A.D.C. (2020). *metan*: An R Package for Multi-Environment Trial Analysis. Methods Ecol. Evol..

[27] dos Santos H.G., Jacomine P.K.T., dos Anjos L.H.C., de Oliveira V.A., Lumbreras J.F., Coelho M.R., de Almeida J.A., de Araujo Filho J.C., de Oliveira J.B., Cunha T.J.F. (2018). Sistema Brasileiro de Classificação de Solos.

[28] Wrege M.S., Steinmetz S., Reisser Júnior C., Almeida I.R. (2011). Atlas Climático da Região Sul do Brasil: Estados do Paraná, Santa Catarina e Rio Grande do Sul.

[29] Bergamaschi H., Melo R.D., Guadagnin M.R., Cardoso L.S., Silva M.D., Comiran F., Brauner P.C. (1970). Boletins Agrometeorológicos da Estação Experimental Agronômica da UFRGS: Série Histórica 1970–2012. Boletim Agrometeorológico: Estação Experimental Agronômica/UFRGS: Eldorado do Sul.

[30] Silveira D.C., Mills A., Sampaio R., Longhi J., Amaral E.C., Ávila V.S., Weiler R.L., Brunes A.P., Simioni C., Dall’Agnol M. (2025). Correlation and path analysis based on multi-trait BLUP as selection criteria for forage in *Paspalum nicorae* Parodi. Rev. Bras. De Zootec. Braz..

[31] Comissão de Química e Fertilidade do Solo (2016). Manual de Calagem e Adubação para os Estados do Rio Grande do Sul e de Santa Catarina.

[32] Brasil (2009). Ministério da Agricultura, Pecuária e Abastecimento. Regras para Análise de Sementes.

[33] Rao C.R. (1973). Linear Statistical Inference and Its Applications.

[34] Dempster A.P., Laird N.M., Rubin D.B. (1977). Maximum Likelihood from Incomplete Data via the EM Algorithm. J. R. Stat. Soc. Ser. B Methodol..

[35] Pimentel-Gomes F. (1991). Índice de Variação: Um Substituto Vantajoso do Coeficiente de Variação. Circ. Téc. IPEF.

[36] Resende M.D.V. (2007). Matemática e Estatística na Análise de Experimentos e no Melhoramento Genético.

[37] Olivoto T., Nardino M. (2021). MGIDI: Toward an Effective Multivariate Selection in Biological Experiments. Bioinformatics.

[38] R Core Team (2025). R: A Language and Environment for Statistical Computing.

[39] Resende M.D.V. (2016). Software Selegen-REML/BLUP: A Useful Tool for Plant Breeding. Crop Breed. Appl. Biotechnol..

[40] Cruz C.D. (2016). Genes Software—Extended and Integrated with the R, Matlab and Selegen. Acta Sci. Agron..

[41] Sebbenn A.M., Siqueira A.C.M.d.F., Kageyama P.Y., Machado J.A.R. (1998). Parâmetros Genéticos na Conservação da Cabreúva—*Myroxylon peruiferum* L.F. Allemão. Sci. For..

[42] Vencovsky R., Paterniani E. (1987). Melhoramento e Produção de Milho no Brasil.

[43] Massaro R.A.M., Bonine C.A.V., Scarpinati E.A., Paula R.C. (2010). Viabilidade de Aplicação da Seleção Precoce em Testes Clonais de *Eucalyptus* spp.. Cienc. Florest..

[44] Valadares N.R., Fernandes A.C.G., Rodrigues C.H.O., Guedes L.L.M., Magalhães J.R., Alves R.A., de Andrade V.C., Azevedo A.M. (2022). Estimation of Genetic Parameters and Selection Gains for Sweet Potato Using Bayesian Inference with *A Priori* Information. Acta Sci. Agron..

[45] Resende M.D.V., Duarte J.B. (2007). Precisão e Controle de Qualidade em Experimentos de Avaliação de Cultivares. Pesqui. Agropecu. Trop..

[46] Oliveira E.J., Santana F.A., Oliveira L.A., Santos V.S. (2014). Genetic Parameters and Prediction of Genotypic Values for Root Quality Traits in Cassava Using REML/BLUP. Genet. Mol. Res..

[47] Azevedo A.M., Andrade Júnior V.C., Fernandes J.S.C., Pedrosa C.E., Oliveira C.M. (2015). Desempenho Agronômico e Parâmetros Genéticos em Genótipos de Batata-Doce. Hortic. Bras..

[48] Falconer D.S., Mackay T.F.C. (1996). Introduction to Quantitative Genetics.

[49] Miranda D.P., Ramos H.C.C., Santa-Catarina R., Vettorazzi J.C.F., Santana J.G.S., Poltronieri T.P.S., Pirovani A.A.V., Azevedo A.O.N., Duarte R.P., Rodrigues A.S. (2022). Topcross Hybrids in Papaya (*Carica papaya* L.): Evaluation of the Potential for Increasing Fruit Quality in New Cultivars. Arch. Agron. Soil Sci..

[50] Fischer S., Möhring J., Schön C.C., Piepho H.P., Klein D., Schipprack W., Utz H.F., Melchinger A.E., Reif J.C. (2008). Trends in Genetic Variance Components during 30 Years of Hybrid Maize Breeding at the University of Hohenheim. Plant Breed..

[51] Gonçalves Z.S., Lima L.K.S., Soares T.L., Souza E.H., Jesus O.N. (2021). Leaf Anatomical Aspects of CABMV Infection in *Passiflora* spp. by Light and Fluorescence Microscopy. Australas. Plant Pathol..

[52] Rosado R.D., Rosado L.D., Borges L.L., Bruckner C.H., Cruz C.D., Santos C.E. (2019). Genetic Diversity of Sour Passion Fruit Revealed by Predicted Genetic Values. Agron. J..

[53] Latheef A., Pugalendhi L., Rani A., Jeyakumar P., Kumar M., Devi M. (2022). Genetic Analysis of Mango (*Mangifera indica* L.) Genotypes for Year-Round Flowering and Yield Characters. Madras Agric. J..

[54] Baker R.J. (2020). Selection Indices in Plant Breeding.

[55] Donald C.M. (1968). The Breeding of Crop Ideotypes. Euphytica.

